# Epidemiology of fractures in adults of African ancestry with diabetes mellitus: A systematic review and meta-analysis

**DOI:** 10.1016/j.bone.2024.117133

**Published:** 2024-05-22

**Authors:** Simon C. Zhang, Tessa Makebeh, Jakub Mesinovic, Kevin Djopseu, Catherine Martin, Li-Yung Lui, Peggy M. Cawthon, Andrea L.C. Schneider, Joseph M. Zmuda, Elsa S. Strotmeyer, Anne Schafer, Peter R. Ebeling, Roger M. Zebaze

**Affiliations:** a Department of Medicine, School of Clinical Sciences, Monash University, Clayton, Victoria, Australia; b ZEZE co, Yaoundé, Cameroon; c Institute for Physical Activity and Nutrition, School of Exercise and Nutrition Sciences, Deakin University, Burwood, Australia; d School of Public Health and Preventive Medicine, Monash University, Clayton, Victoria, Australia; e Research Institute, California Pacific Medical Center, San Francisco, CA, USA; f Department of Epidemiology and Biostatistics, University of California, San Francisco, CA, USA; g Department of Neurology, University of Pennsylvania Perelman School of Medicine, Philadelphia, PA, USA; h Department of Biostatistics, Epidemiology, and Informatics, University of Pennsylvania Perelman School of Medicine, Philadelphia, PA, USA; i Department of Epidemiology, School of Public Health, University of Pittsburgh, Pittsburgh, PA, USA; j Endocrine Research Unit, San Francisco Veterans Affairs Health Care System, San Francisco, USA.; k Department of Medicine, University of California, San Francisco, CA, USA; l Department of Endocrinology, Monash Health, Clayton, Victoria, Australia

**Keywords:** Bone, Diabetes, Fracture, Epidemiology, African, Meta-analysis

## Abstract

Diabetes mellitus (DM) is associated with increased fracture risk in White adults. However, the impact of DM on fractures in Black adults is unknown. This systematic review and meta-analysis investigated the association between DM and fractures in adults of African ancestry. MEDLINE, Scopus, CINAHL and Embase databases were searched from their inception up to November 2023 for all studies in the English language investigating the epidemiology of fractures (prevalence, incidence, or risk) in Black men and women (age ≥ 18 years) with type 1 or type 2 DM. Effect sizes for prevalence of previous fractures (%) and incident fracture risk (hazard ratio [HR]) were calculated using a random-effects model on Stata (version 18.0). There were 13 eligible studies, of which 12 were conducted in Black adults from the United States, while one was conducted in adults of West African ancestry from Trinidad and Tobago. We found no fracture data in Black adults with DM living in Africa. Five studies were included in a meta-analysis of incident fracture risk, reporting data from 2926 Black and 6531 White adults with DM. There was increased risk of fractures in Black adults with DM compared to non-DM (HR = 1.65; 95 % confidence interval [CI]: 1.14, 2.39). The risk of fractures was also higher in White adults with DM compared to non-DM (HR = 1.31; 95 % CI: 1.06, 1.61) among these studies. Five studies were included in a meta-analysis of fracture prevalence, of which three also reported fracture prevalence in White adults. There were 175 previous fractures among 993 Black adults with DM and 384 previous fractures among 1467 White adults with DM, with a pooled prevalence of 17.5 % (95 % CI: 15.4, 19.6) and 25.8 % (95 % CI: 4.8, 46.8), respectively. Our results indicate a high burden of fractures in Black adults with DM.

## 1. Introduction

Diabetes mellitus (DM) and fragility fractures are major public health challenges associated with significant morbidity and mortality [1–4]. Of particular concern is the increasing burden of both conditions in low- and middle-income countries, due to epidemiological transitions such as increased longevity and lifestyle changes driven by rapid urbanisation [5,6]. In Africa alone, the prevalence of type 2 diabetes mellitus (T2DM) is expected to rise by 143 % from 19.4 million in 2019 to 47.1 million in 2045 – far greater than the estimated global average increase of 51 % within the same timeframe [7]. Meanwhile, there is a continued lack of awareness regarding the impact of fragility fractures in Africa, despite the increasing incidence of fractures observed in this population [8–12].

Macrovascular and microvascular complications secondary to DM are well-established in clinical practice. However, the skeletal consequences of long-term hyperglycaemia remain understudied. Previous meta-analyses indicate that both type 1 diabetes mellitus (T1DM) and T2DM are associated with an increased risk of hip and non-vertebral fractures [13–16], and that bone mineral density (BMD) is decreased in T1DM but paradoxically elevated in T2DM [16]. Nevertheless, the majority of observational studies in these reviews primarily pertain to White populations [14], despite the higher prevalence of DM in other racial and ethnic groups such as Black adults [17]. Among large multi-ethnic cohorts in the United States, results comparing fracture risk in Black and White adults in DM are conflicting, with some studies reporting increased risk of fracture [18,19] or no discernible disparities between the two ethnicities [20]. The generalisability of these results is further limited by the relatively small sample sizes of Black individuals [18–20].

At present, there is a paucity of data on bone disease in adults of African ancestry with DM, especially among the 1.5 billion people living on the African continent. As diabetes prevalence continues to escalate in Africa, it is expected to concurrently accentuate the public health burden of low-trauma fractures, imparting significant health and economic consequences. Hence, we conducted a systematic review and meta-analysis to define the burden of bone disease in adults of African Ancestry with T1DM and T2DM, with a focus on epidemiological aspects including prevalence, incidence, and risk of fracture in this population.

## 2. Methods

This systematic review was performed and reported in accordance with the Preferred Reporting Items for Systematic Reviews and Meta-Analyses (PRISMA) guidelines [21]. The protocol was registered in PROSPERO on July 6, 2023 (CRD42023439387).

### 2.1. Search strategy

We conducted a systematic search of four electronic databases including MEDLINE, Scopus, CINAHL and Embase from their inception to June 10, 2023 using keywords relating to diabetes (diabet*), bone fracture (fracture*, osteoporo*, fragil*) and adults of African descent (Africa*, Black*, and the ‘African search filter’ [22] which lists the individual names of all African countries including truncated terms). The search was limited to reports published in the English language. An updated search was performed on November 22, 2023. The full search strategy is available in Supplementary Table 1.

### 2.2. Eligibility criteria

The following eligibility criteria were used in the selection process of the review:

1. 1Study population: Men and women (age ≥ 18 years) of African ancestry as defined by Black adults residing both within the African continent and continents outside Africa. Studies were excluded if they did not provide outcomes specific to the target population.
2. Exposure: History of DM (type 1 or type 2) determined by patient self-report, physician diagnosis, use of diabetes medications, or blood tests (hemoglobin A1c, fasting glucose, oral glucose tolerance test). Studies were excluded if the definition of DM was not clearly defined, or if outcomes were reported in individuals without T1DM or T2DM (i.e. impaired glucose tolerance or gestational diabetes).
3. Outcomes: The epidemiology of fractures including prevalence of previous fractures in Black adults with DM, incidence of fractures in Black adults with DM, and risk of fractures in Black adults with DM compared to Black adults without DM.
4. Study type: All studies (case report/series, cross-sectional, case-control, cohort, etc.) and conference abstracts were included. Systematic reviews and review papers were excluded.

### 2.3. Study selection

Two reviewers (SZ and TM) independently screened all titles and abstracts imported into Covidence to exclude non-relevant records. Full texts of all remaining studies were then obtained and underwent full-text screening based on the inclusion and exclusion criteria. Discrepancies between the two reviewers during any stage were resolved through discussion. The reference list of eligible studies identified after full-text screening, as well as the references cited in similar systematic reviews related to the study topic [13–15], were manually reviewed to identify pertinent studies. Keywords from the initial search strategy were also used to search online databases such Google Scholar and Africa Journals Online, where the first 100 studies generated in the results from each database were reviewed to assess their eligibility for inclusion. Moreover, we contacted the authors of several studies for data relevant to our research question (Supplementary Table 2).

### 2.4. Data extraction

For each study in the systematic review, the following data were gathered: first author's surname, year of publication, country, study design, study cohort, type of diabetes (type 1, type 2, or both), age at enrolment, sex, race, mean follow up time and standard deviation (cohort studies), and fracture definition/sites analysed. In both Black and White adults (where available), further data collected include sex (% female), number of fractures in adults with DM, total number of adults with DM, prevalence of previous fractures (%), and/or risk estimates of fracture (relative risk [RR], hazard ratio [HR]) with corresponding confidence intervals (CI) and controlled variables in multivariable analyses. For each study reporting risk of fracture, the risk estimate that reflects the greatest degree of control for potential confounding were extracted. All data were collected in a Microsoft Excel spreadsheet by a single reviewer (SZ), which was subsequently checked by the second reviewer (TM).

### 2.5. Quality assessment

Eligible articles were assessed independently by the two primary reviewers (SZ and TM) for their methodological quality using the Joanna Briggs Institute (JBI) critical appraisal checklists specific to the study design [23]. Prevalence and cohort studies were classified as low risk of bias if they scored >70 %, moderate risk of bias if they scored between 50 % and 69 %, and high risk of bias if they scored below 50 % [24].

### 2.6. Statistical analysis

Random-effects model with restricted maximum likelihood estimation was used to obtain the effect sizes for prevalence of previous fractures (%) and risk of incident fractures (HR) with 95 % confidence intervals in Black adults with DM compared to non-DM. The civartolerance option was required to relax the tolerance for non-symmetrical confidence interval as symmetry was not corrected with log-transformation of the HR and 95 % CIs. The confidence intervals were then backtransformed when generating the graph which may have introduced minor variations to some of the original CI values. The Hartung-Knapp-Sidik-Jonkman (HKSJ) approach was employed to estimate the variance of the pooled effect in all meta-analyses with <5 studies (k) [25]. This method mitigates type 1 error rates compared with the DerSimonian-Laird method when the number of included studies is small [26]. In statistical analyses comparing prevalence and risk of fractures between Black and White adults, meta-regression could not be performed as this is not recommended when k < 10 [27]. As a result, subgroup analyses of prevalence and risk of fractures in Black and White adults with DM using the random-effects model with HKSJ method was employed instead, with test of group differences used to determine statistical significance of differences between the two ethnicities. All analyses were carried out using the Meta commands in Stata BE version 18.0 (StataCorp, USA).

Due to the small number of studies in the meta-analysis, only two important variables – age and sex – were considered in subgroup analyses. Analysis by sex was divided into three groups: 1) studies with females only; 2) studies with males only; and 3) studies with males and females. Meanwhile, analysis by age was divided into three groups according to the age of enrolment in studies reporting prevalence: 1) age 40–93 years; 2) age 56–66 years; and 3) age ≥ 65 years. In studies reporting fracture risk, age was divided as follows: 1) age 45–64 years; 2) age 50–79 years; and 3) age ≥ 65 years. Statistical heterogeneity between studies was measured using Cochran's Q test. We considered I^2^ < 40 % as reflecting minimal heterogeneity, 30–60 % as reflecting moderate heterogeneity and 50–90 % as reflecting substantial heterogeneity [27]. We were unable to assess publication bias due to the limited number of studies (k <10) [28]. *P* values <0.05 were considered statistically significant. All statistical tests were two-sided.

## 3. Results

**Fig. 1.**
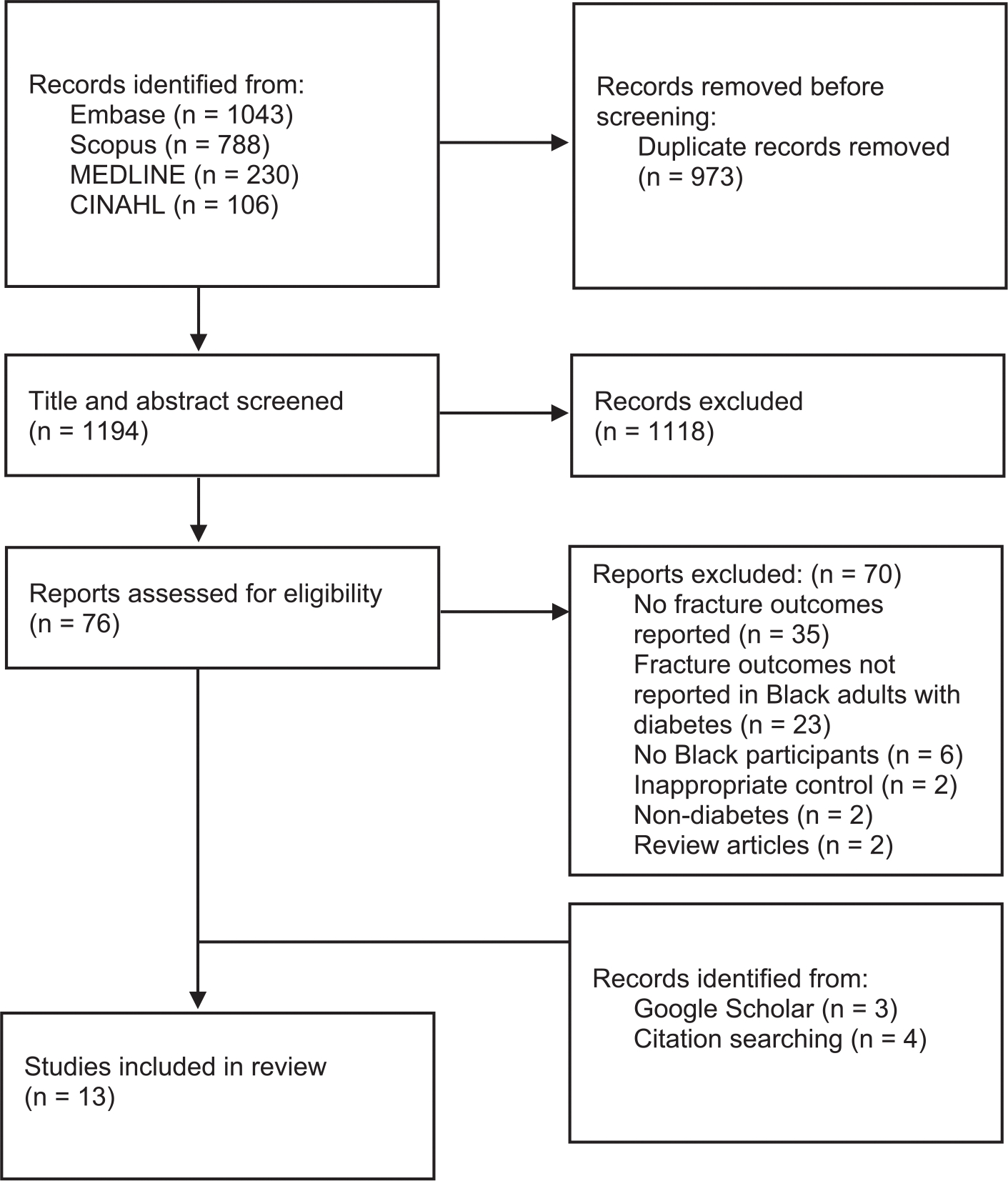
PRISMA flow diagram of literature search and selection.

The initial search identified 2167 studies, of which 973 duplicate records were automatically removed when imported into Covidence. Of the 1194 unique studies, 1118 studies were excluded following evaluation of titles and abstracts. We retrieved the full-text of 76 studies that met the inclusion criteria, and a further 70 studies were excluded after full-text assessment for the following reasons: 35 studies did not report fracture outcomes; 23 studies included fracture outcomes but did not report this data in Black adults with DM; six studies did not include Black participants; two studies used hypertensive patients as the control group; two studies reported outcomes in participants with insulin resistance or impaired glucose tolerance only; and two studies were review articles (Supplementary Table 3). A manual search of the reference lists of the six eligible studies [18–20,29–31] yielded a further four relevant studies that met our inclusion criteria [32–35]. Another three studies were identified based on our eligibility criteria from Google Scholar [36–38]. Altogether, a total of 13 studies were included in this review, and the search process is presented in the PRISMA diagram (Fig. 1).

We found no studies that reported the prevalence, incidence, or risk of fractures among Black adults with DM living in Africa. Outside of Africa, one study investigated the prevalence of previous fractures in a cohort of adults of West African ancestry, albeit only in men [29]. Other studies were performed in multi-ethnic cohorts which conducted subgroup analyses in African Americans, of which only data relating to the prevalence of previous fractures [30–34,36] and risk of fractures [18–20,34,35,37,38] were available for analysis. Although multiple studies used duplicate community cohorts, there was no overlapping cohorts and patients in meta-analysis.

### 3.1. Quality assessment

All studies employed appropriate methodologies and analyses on critical appraisal (Supplementary Table 4). Among prevalence studies, four studies used either population-based listings or convenience sampling methods to recruit participants [30,32,34,36], three studies included small sample sizes of <100 adults with DM [31,32,34], and the response rates of several studies could not be ascertained [29,33,34]. Prevalence and cohort studies used valid methods for identification of diabetes and previous/incident fractures, although the quality of how these exposures/outcomes were recorded varied. For example, one study relied only on patient self-report of medical and fracture history [29], while other studies confirmed diabetes by blood tests [18–20,30,31,33,34,37,38] and/or fractures via radiographic report or physician diagnosis codes [20,30–32,34–38] in addition to patient self-report.

All cohort studies compared participant baseline characteristics effectively, had adequate follow-up durations of at least 4.5 years, adjusted for important confounding variables, and reported appropriate statistical methods and analysis, although only one study accounted for competing risks (fracture vs. death) to evaluate the impact of survival bias [37]. One study excluded participants with prior fractures at baseline [18], two studies explored reasons for loss to follow-up [18,35], and two studies described strategies to address for incomplete follow-up [18,19].

### 3.2. Prevalence of previous fractures: Study characteristics

There were seven studies (Table 1) that reported the prevalence of previous fractures in Black adults with DM [29–34,36], of which four studies also reported this outcome in White adults with DM [32–34,36]. Study sample sizes ranged from 22 to 675 Black participants with DM and 48 to 173 White participants with DM. Three studies were cross-sectional [29,31,32] and four studies were prospective [30,33,34,36]. Six studies were performed in the United States [30–34,36] and reported outcomes in African American adults, whilst one study was performed in Trinidad and Tobago [29] and reported outcomes in Caribbean males of West African ancestry. There was a total of six different community cohorts: The Tobago Bone Health Study [29]; Health, Aging, and Body Composition (Health ABC) Study [33]; Study of Women's Health Across the Nation (SWAN) [31]; The Osteoporotic Fracture in Men (MrOS) Study [34]; African American-Diabetes Heart Study (AA-DHS) [30]; and the Study of Osteoporotic Fractures (SOF) [32,36]. Two studies described outcomes in men only [29,34], three studies described outcomes in women only [31,32,36], and two studies described outcomes in both men and women [30,33]. Three studies included exclusively participants with T2DM [30,31,33], one study included participants with both T1DM and T2DM [34], and three studies did not specify the subtype of DM assessed [29,32,36]. Age of enrolment among the community cohorts ranged from 30 years to 93 years. The definition of fracture prevalence varied across the different studies. The five studies included in the meta-analysis defined fractures as follows: any previous fractures [29]; previous fractures > age 45 years [33]; previous fracture > age 20 years (excluding hand, foot and face fractures) [31]; previous fractures > age 50 years [36]; and previous fractures ≥ age 50 years [34]. Two studies [30,32] were not included in the meta-analysis as they investigated prevalent vertebral fractures only.

### 3.3. Prevalence of previous fractures (excluding vertebral fractures): Meta-analysis results

**Fig. 2.**
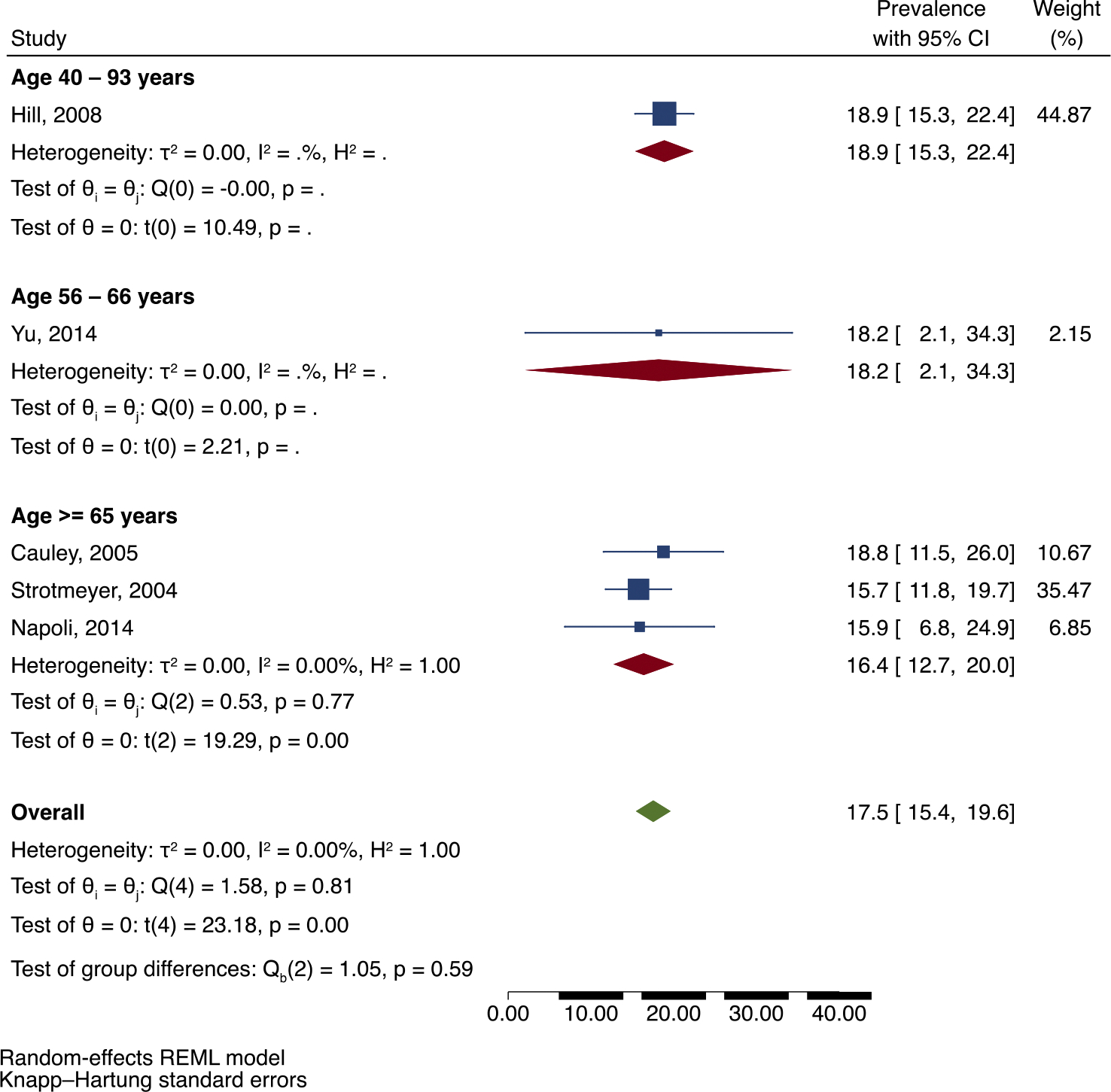
Prevalence of previous fractures (% percent) in adults of African ancestry with DM with subgroup analyses by age category.

**Fig. 3.**
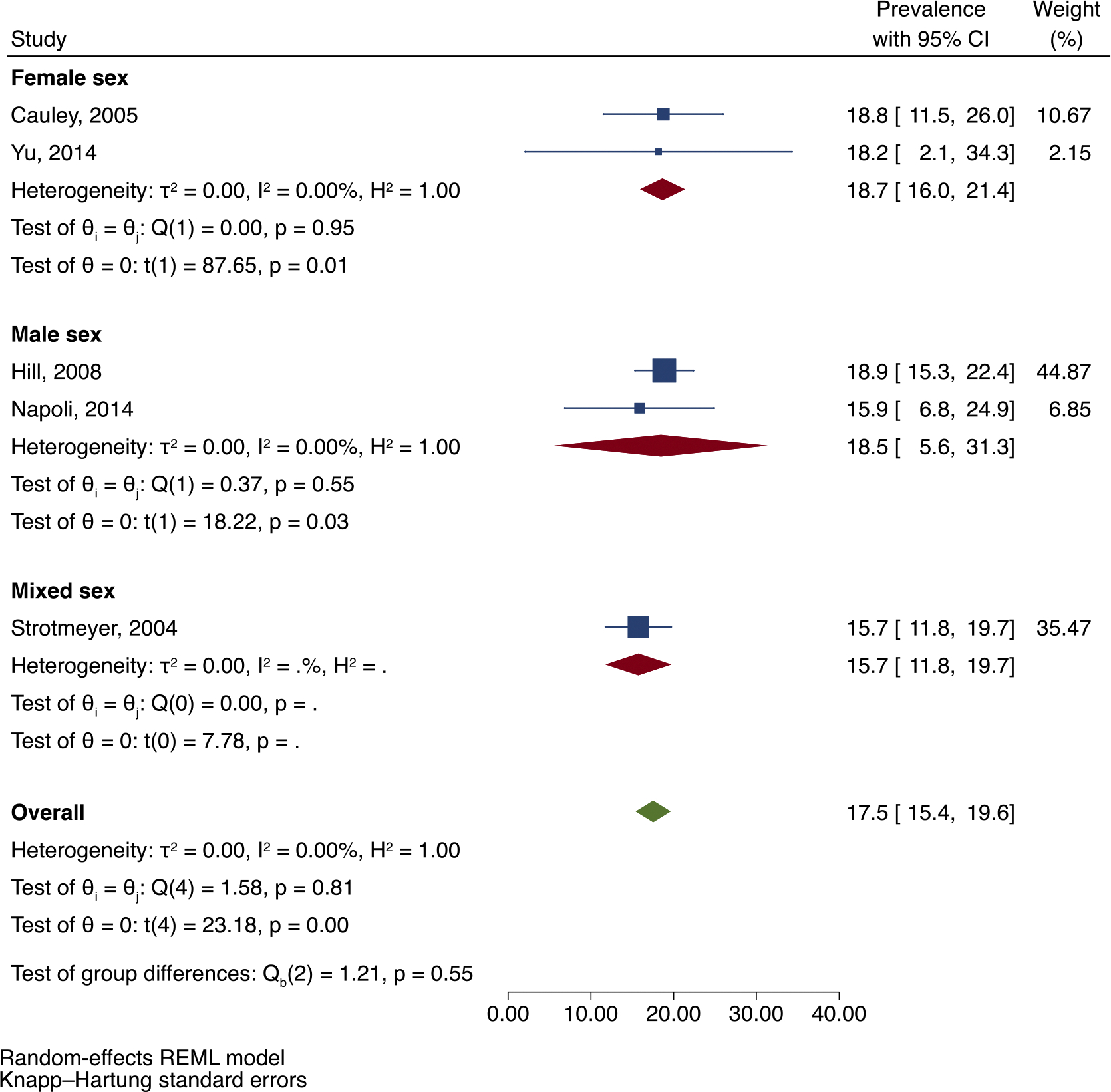
Prevalence of previous fractures (% percent) in adults of African ancestry with DM with subgroup analyses by sex.

In meta-analysis of five studies reporting a total of 175 fractures among 993 Black adults with DM, pooled prevalence of all previous fractures was 17.5 % (95 % CI: 15.4, 19.6). Analysis by age category and sex can be seen in Figs. 2 and 3 respectively, although this was limited by the small number of studies. Fig. 2 shows that the pooled prevalence of previous fractures among then age category ≥65 years was 16.4 % (95 % CI: 12.7, 20.0), while there was only one study in the age category 56–66 years and one study in the age category 40–93 years. Fig. 3 shows that in analysis by sex, the prevalence of previous fractures was similar between men (18.5 %; 95 % CI: 5.6, 31.3) and women (18.7 %; 95 % CI: 16.0, 21.4) with DM, while there was only one study in the mixed-sex group. There was minimal heterogeneity between the five studies in this analysis (I^2^ = 0.00; *p* = 0.81).

**Fig. 4.**
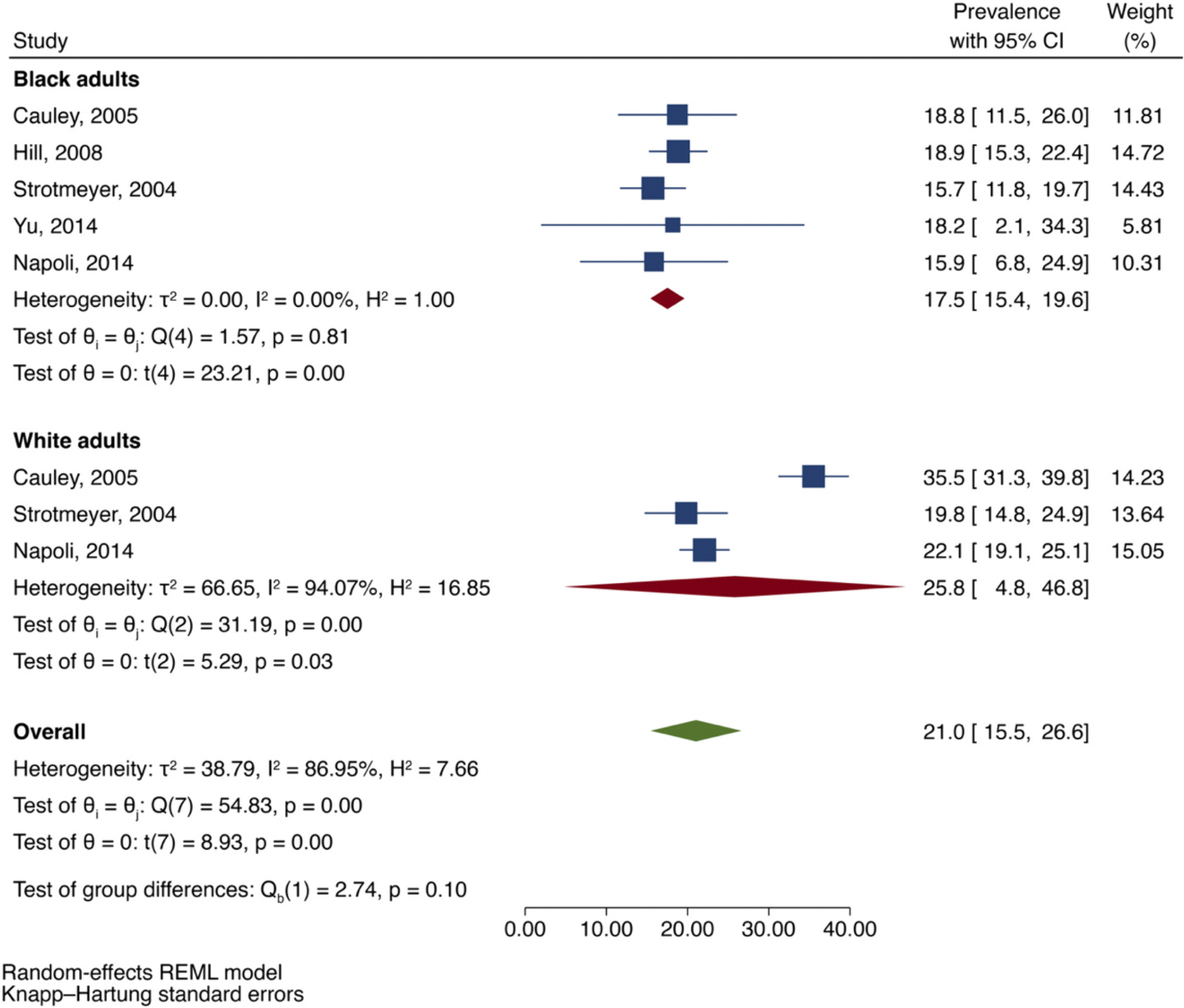
Prevalence of previous fractures (% percent) in Black adults with DM compared to White adults with DM.

Among the five studies in the meta-analysis, three studies reported the prevalence of previous fractures in both Black and White adults with DM [33,34,36]. There were 384 fractures in 1467 White adults with DM among the three studies. Compared to Black adults with DM (prevalence = 17.5 %; 95 % CI: 15.4, 19.6), the prevalence of previous fractures in White adults with DM was 25.8 % (95 % CI: 4.8, 46.8). However, there was substantial heterogeneity (I^2^ = 86.95; *p* = 0.00) and statistical tests of group differences were not significant (*p* = 0.10), as demonstrated in Fig. 4.

### 3.4. Prevalence of vertebral fractures

Two studies reported 86 vertebral fractures among 755 African Americans with DM, with an average prevalence of 11.3 %. Lenchik et al. [30] found that in 675 male and female participants with T2DM over the age of 30 years from the AA-DHS cohort, the prevalence of vertebral fractures (diagnosed using sagittal CT images obtained during the initial study visit) was 11.0 %. In Cauley et al. [32], the authors reported that the prevalence of vertebral fractures (identified from lateral spine radiographs using vertebral morphometry) was 15.0 % among 80 African American women with DM aged 70 years or above, compared to 16.1 % among 515 White women with DM.

### 3.5. Risk of incident fractures: study characteristics

Table 2 shows the characteristics of the seven studies reporting incident fracture risk in Black adults with DM, while all studies also reported this outcome in White adults with DM [18–20,34,35,37,38]. Study sample sizes ranged from 63 to 1774 Black adults with DM and 318 to 4453 White adults with DM. All studies were performed in the United States from five different community cohorts: The Health, Aging, and Body Composition (Health ABC) Study [20,38]; The Osteoporotic Fracture in Men (MrOS) Study [34]; the Atherosclerosis Risk in Communities (ARIC) Study [37]; The Women's Health Initiative (WHI) [19,35]; and The third National Health and Nutrition Examination Survey (NHANES III) and NHANES 1999–2004 [18]. Two studies described outcomes in women only [19,35], one study described outcomes in men only [34], and four studies described outcomes in both men and women [18,20,37,38]. Three studies included participants with T2DM exclusively [19,20,37], two studies included participants with both T1DM and T2DM [18,34], and two studies did not state the type of DM that was assessed [35,38]. Age of enrolment among the community cohorts ranged from 45 years to 79 years. The skeletal fracture sites collected in each study included: non-vertebral fractures [34,38]; any non-skull fractures [18]; all fractures except fingers, toes, face, skull, or sternum [35]; any incident fracture hospitalization [37]; vertebral, shoulder, upper arm, lower arm, wrist, hip, upper leg, lower leg, and foot fractures [19]; and any fractures [20]. All studies controlled for age, while all studies with male and female participants also controlled for sex [18,20,37,38]. Five studies controlled for a variety of variables in addition to age and/or sex [18–20,34,38]. The five studies included in the meta-analysis expressed fracture risk as hazard ratio [18,34,35,37,38], while two studies reported relative risk [19,20].

### 3.6. Risk of incident fractures as hazard ratio: meta-analysis results

**Fig. 5.**
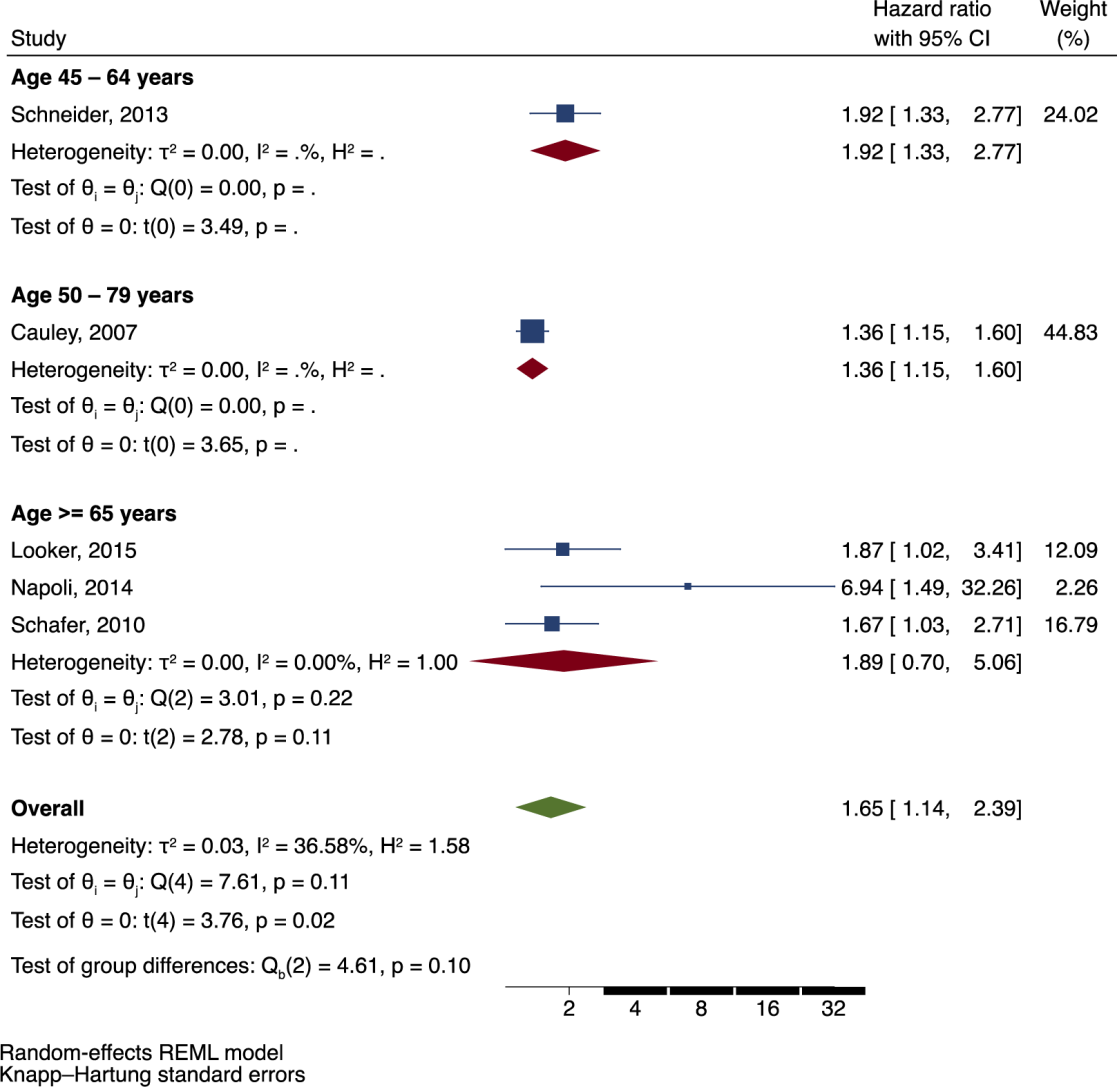
Incident fracture risk in adults of African ancestry with DM with subgroup analyses by age category.

**Fig. 6.**
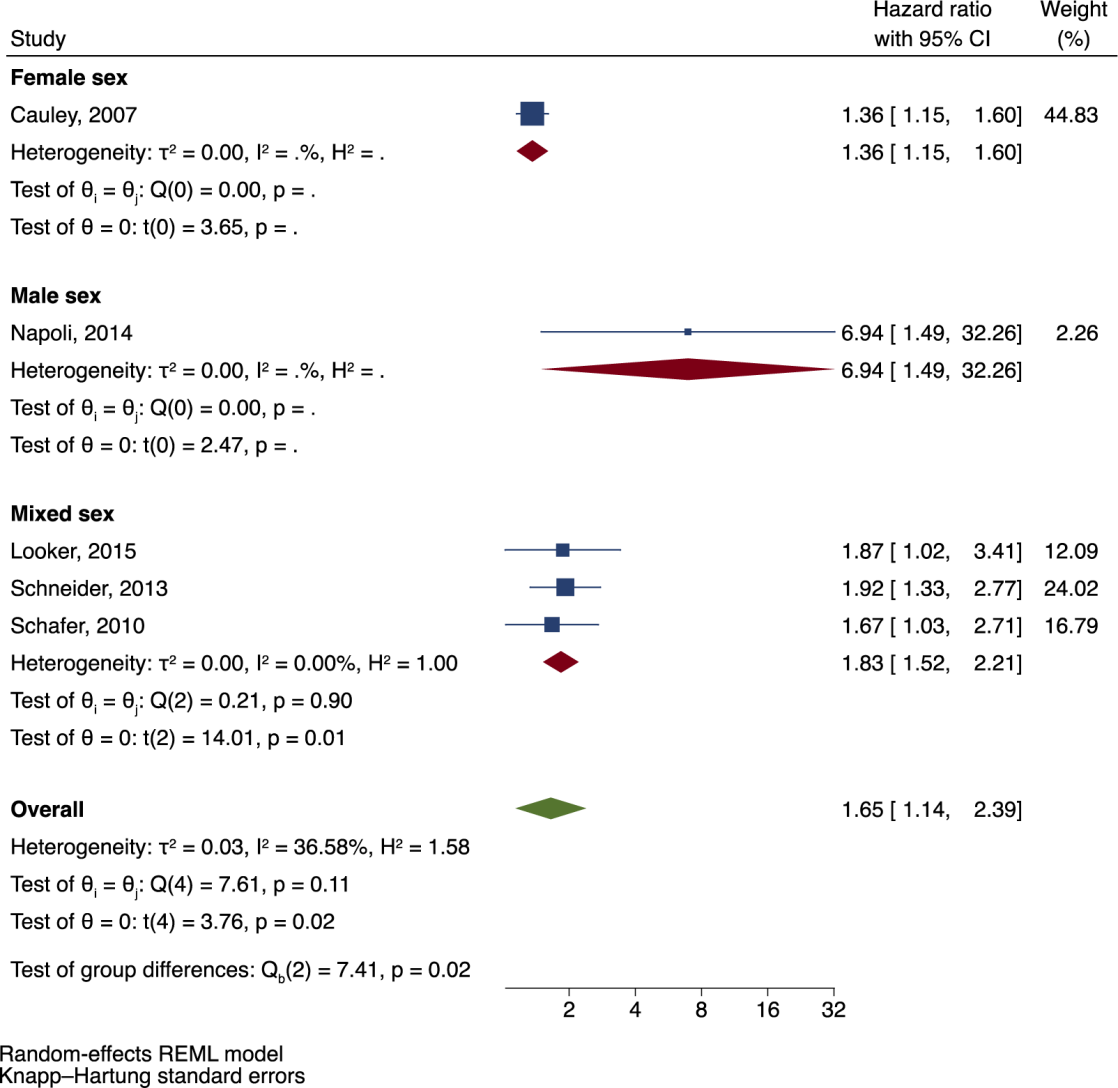
Incident fracture risk in adults of African ancestry with DM with subgroup analyses by sex.

There was a statistically significant increased risk of all fractures among a total of 2926 Black adults with DM compared to Black adults without DM (HR =1.65; 95 % CI: 1.14, 2.39). Moreover, there was also a statistically significant positive association reported in each individual study included in the pooled analysis. Subgroup analyses of fracture risk by age category and sex are presented in Figs. 5 and 6 respectively, although this was limited by the small number of studies in the meta-analysis. The hazard ratio of incident fracture among African American adults with DM in the age category ≥65 years was 1.89 (95 % CI: 0.70, 5.06), while there was only one study in the age category 45–64 years and one study in the age category 50–79 years. In analysis by sex, the hazard ratio of incident fracture in the mixed sex cohort was 1.83 (95 % CI: 1.52, 2.21), while there was one study with male participants only and one study with female participants only. Napoli et al. reported the highest risk of fractures in men from the MrOS cohort compared to other studies (HR = 6.94; 95 % CI: 1.49, 32.26). However, there was a very small number of fractures (*n* = 5) among a limited sample size of men with DM (*n* = 63). Overall, there was moderate heterogeneity between the five studies included in the meta-analysis (I^2^ = 36.58; *p* = 0.11).

**Fig. 7.**
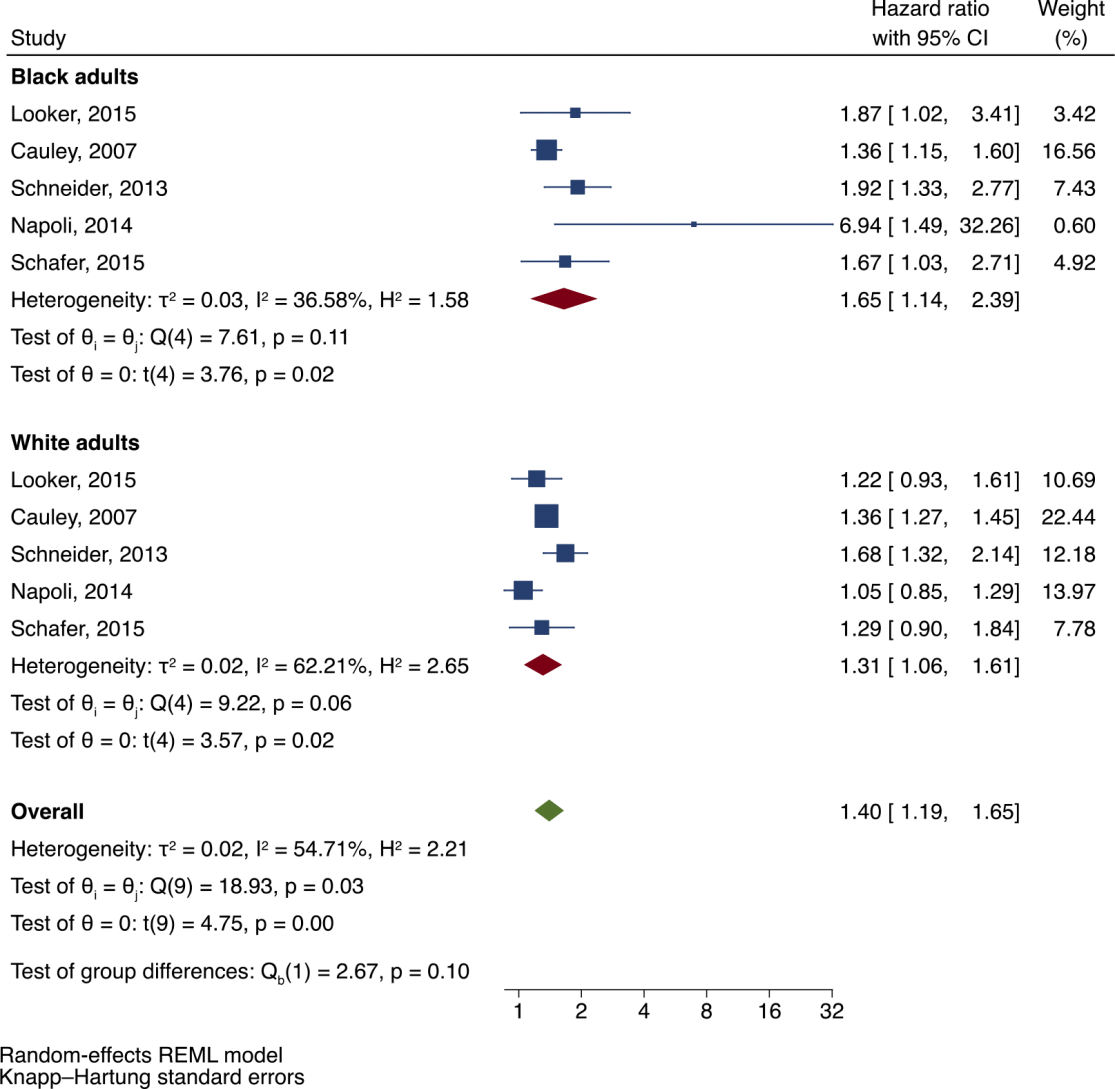
Incident fracture risk in Black adults with DM compared to White adults with DM.

All five studies in this analysis also reported risk of incident fractures in White adults with DM (*n* = 6531), in which there was an elevated risk of fracture (HR = 1.31; 95 % CI: 1.06, 1.61) (Fig. 7). Black adults with DM in the same cohorts had a similar fracture risk (HR = 1.65; 95 % CI: 1.14, 2.39; test for race differences: *P* = 0.10), with moderate heterogeneity between studies (I^2^ = 54.71; *p* = 0.03). Of note, only two out of the five studies reported a statistically significant increased risk of fractures in White adults with DM compared to counterparts without DM [35,37].

## 3.7. Risk of incident fractures as relative risk

When considering relative risk, both Black (RR =1.46; 95 % CI: 0.21. 10.20) and White adults (RR = 1.19; 95 % CI: 0.72, 1.97) with DM had increased risk of fractures compared to Black and White adults without DM, respectively (Supplementary Fig. 1). However, both results were not statistically significant, and there were only two studies included in the analysis. Strotmeyer et al. [20] found a significantly elevated risk of any fractures (RR =1.87; 95 % CI: 1.11, 3.17) in Black adults with T2DM compared those without T2DM in post-hoc analyses. Bonds et al. [19] also reported an increased risk of vertebral and non-vertebral fractures in Black women with T2DM compared to counterparts without T2DM, however, this result was not statistically significant (RR = 1.33; 95 % CI: 1.00, 1.75).

## 4. Discussion

The main findings from this systematic review and meta-analysis indicate that DM is associated with a high prevalence of any previous fractures as well as an elevated risk of incident fractures among adults of African ancestry. There was a 65 % increase in the risk of fractures among Black adults with DM compared to without DM, and a 31 % increased risk in White adults with DM compared to counterparts without DM. Moreover, the prevalence of previous fractures appeared to be lower in Black adults with DM (17.5 %) compared to White adults with DM (25.8 %), although there was a lack of statistically significant group differences between the two ethnicities.

Ostensibly, there is no published literature characterising the epidemiology of fractures among Black adults with DM living in the African continent. Regardless, our search revealed two studies from Africa that assessed the risk of DM as a comorbidity among adults presenting with fractures. Dela et al. [39] performed a prospective study across 28 hospitals in South Africa and reported that the prevalence of DM was 29.6 % among 202 subjects who presented with acute fragility hip fractures. However, the demographic characteristics of the study was diverse, wherein only 39 % of the study population were Black adults [39]. Another prospective study by Baidoo et al. [40] reported that the prevalence of DM was 23.7 % among 76 Black adults who presented with proximal femur fractures at a major tertiary hospital in Ghana. Despite the high prevalence of DM as a comorbidity in femoral fractures, these outcomes do not address our primary research question. In studies conducted outside of Africa, literature investigating the prevalence, incidence, or risk of fractures in Black adults was limited. This knowledge gap is particularly concerning considering the increasing prevalence and burden of both DM and fragility fractures in Africa and in the Black community in general [6,7].

Our study reported no group differences in the prevalence of previous fractures between Black and White adults with DM, although all individual studies reported a higher prevalence in the White population with DM. This is despite the possibly higher risk of incident fractures in Black adults with DM. One explanation for this may be the higher absolute fracture risk in non-diabetic White adults compared to other racial/ethnic groups, including Black adults [41]. Nevertheless, with our reported prevalence of previous fractures (17.5 %), we estimate that over 3 million of the 19.4 million adults currently living with DM in Africa [7] may have experienced at least one previous fracture. This makes bone disease in this population a significant yet neglected public health problem. However, these generalisations are made with the caveat that included studies in this review were carried out mostly in African American cohorts, while one study was conducted in Caribbean males on the island of Tobago; the two populations have a European admixture of approximately 25 % [42] and 6 % [43] respectively.

Our data are consistent with previous meta-analyses exploring the risk of fractures in DM, although the results from those studies predominantly pertain to White adults. Moayeri et al. [15] analysed 30 case-control and cohort studies between 1980 and May 2016 and reported a significant association between T2DM and fracture risk at both non-vertebral and vertebral sites (RR = 1.05, 95 % CI: 1.04, 1.06). However, only five of the 30 studies [18–20,34,37] included multi-ethnic cohorts reporting results in Black adults. We reported a higher risk of fracture in Black adults (HR = 1.65; 95 % CI: 1.14, 2.39), although we included fewer studies, participants with T1DM and evaluated time-to-event data as opposed to dichotomous data. Indeed, there is evidence showing that T1DM confers a higher risk of hip fractures compared to T2DM [13,14,16], although evidence regarding non-vertebral fractures is equivocal [13]. When considering non-vertebral sites only, Janghorbani et al. [14] found a statistically significant association between T2DM and risk of non-vertebral fractures (RR = 1.2; 95 % CI: 1.01, 1.5). Similarly, Vilaca et al. [13] examined the risk of non-vertebral fractures in adults with both T1DM and T2DM but revealed a significantly elevated risk in adults with T2DM only (T1DM RR = 1.92, 95 % CI: 0.92, 3.99; T2DM RR = 1.19, 95 % CI: 1.11, 1.28). Both reviews had little information available regarding the relationship between DM and fractures in Black adults. In our review, Schafer et al. [38] and Napoli et al. [34] both considered non-vertebral fractures only and reported significant increases in fracture risk in Black adults with DM (HR = 1.67; 95 % CI: 1.03, 2.71 and HR = 6.94; 95 % CI: 1.49, 32.2 respectively), although these studies were limited by the low number of participants with DM (*n* = 340 and *n* = 63, respectively).

Our review further demonstrates a higher risk of fracture in Black adults with DM compared to White adults with DM, however race differences were not statistically significant. Three studies found no significant differences between DM with risk of fracture in analyses that compared Black and White adults [20,34,37]. Conversely, Looker et al. [18] reported that the risk of any non-skull fractures in DM may be higher in non-Hispanic Black adults (HR = 1.87; 95 % CI 1.02, 3.40) compared with non-Hispanic White adults (HR = 1.22; 95 % CI: 0.93, 1.61). Similarly, data from the WHI-OS [19] suggest a somewhat higher risk of any fractures in non-Hispanic Black women with DM (RR = 1.33, 95 % CI: 1.00, 1.75) compared to non-Hispanic White women with DM (RR = 1.18; 95 % CI: 1.08, 1.29). It is unclear whether our findings in African American adults with DM extend to increased fracture risk in Black adults with DM globally. Such comparisons are inherently challenging as fracture probability is strongly influenced by a combination of genetic, environmental and lifestyle differences which vary significantly across different regions in the world [44]. Indeed, there is a 10-fold range in probability of hip fractures globally, representing a greater difference in the incidence observed between sexes within a single country [45]. Kanis et al. reported that the 10-year probability of a major osteoporotic fracture in Black men and women aged 65 years with a prior fragility fracture in the US was higher compared to Black counterparts living in South Africa [45]. Nevertheless, this may be in part due to the lower mortality risks following fractures in those from the US [45]. A systematic review of 33 countries/regions found that the rate of hip fractures was highest in countries/regions that were situated either far north (Scandinavia and Canada) or far south (Argentina) of the equator [43]. Whether this translates to increased fracture risk in Black adults living in these regions, however, is unknown. The mechanisms underlying increased bone fragility in adults of African ancestry also remains poorly understood, and it is likely that factors such as socioeconomic status, a key indicator of access to healthcare and health-related behaviors, may play an important role. A 1995 population-based study investigating racial differences associated with diabetes reported that the excess prevalence of diabetes in African American adults could not be fully accounted by biological differences such as obesity and central adiposity alone. Rather, differences in diabetes prevalence compared to White adults was greatest among individuals with lower socioeconomic status [46]. The role of socioeconomic factors in diabetes risk has since been supported by multiple further studies [47–49], as has its contribution to fracture risk [50,51]. Hence, future studies should duly consider the complex interplay between clinical, demographic, and socioeconomic factors in the evaluation of fracture rates in diabetes across different racial groups.

Existing literature suggest that Black adults living in Africa appear to have a lower incidence of fractures than other ethnic groups [39,52], however outcomes following fragility fractures are often worse in Black individuals [11,53,54]. The lower fracture rates in Black adults may be due to higher overall bone density and lower BMD loss with age compared to other ethnicities [41]. However, this explanation is complicated by the paradox in T2DM, where there is increased bone fragility despite normal or even increased BMD [16,55], suggesting that BMD cannot explain the increased bone fragility in individuals with T2DM. Results from microarchitecture studies in T2DM have also reported conflicting findings [56–58], indicating that bone fragility observed in T2DM does not appear to be entirely explained by bone structure [59]. At a molecular and cellular level, mechanisms such as formation and accumulation of advanced glycation end products (AGEs), osteocyte senescence, and inhibition of Wnt signalling, among others, may contribute to increased bone fragility [60]. These alterations in cellular and molecular properties of bone cannot be adequately captured using a quantification method of bone mass such as dual-energy x-ray absorptiometry (DXA) scans, which are not intended to measure tissue material properties such as fatigue strength and fracture toughness [61]. Other approaches such as the Trabecular Bone Score and high-resolution peripheral quantitative computed tomography (HRpQCT) have attempted to address these limitations by assessing cortical and trabecular microarchitecture, but their improvements in predicting fracture risk are only modest [60]. The diagnostic utility of DXA and HRpQCT machines are further hindered by their limited availability and high operating costs in Africa [12]. This is highlighted by a 2011 International Osteoporosis Foundation report showing the provision of only five DXA scans per million people in Tunisia and 0.6 DXA scans per million in Morocco [62]. In turn, there has been increasing development and use of ethnic-specific Fracture Risk Assessment Tool (FRAX) models in countries such as South Africa [39] and Botswana [52]. Certainly, FRAX tools serve as a promising modality which increases accessibility to bone health assessment without BMD measurement, especially in resource poor countries. However, they do so without appreciating the underlying pathogenesis of fragility fractures and may ultimately underestimate fracture risk [60]. Thus, tools that improve fracture prediction in DM, whilst also being affordable and accessible, are imperative in Africa.

Ineffective load conduction (ILC) secondary to disorganized bone tissue may be an additional contributor to bone fragility in DM [63]. We suggest that the cellular and molecular changes contributing to the aberrant bone microenvironment in DM may produce disorganized tissue. For example, accumulation of AGEs may lead to premature aging of bone tissue by increasing rigidity and rendering collagen structures incompetent, contributing to disorganized bone tissue that is more likely to transfer loads abnormally and lead to tissue damage, microcracking and ultimately bone failure [63]. Therefore, bone fragility in DM is an example of disorganized bone tissue disorder and current challenges in diagnosis and management may stem from evidence suggesting that low bone density may not be the key problem [63]. Given that bone disorganization can be measured with high precision and accuracy from standard X-rays routinely available as described in our recent review [64], this may reduce the need for more expensive technologies such as DXA or HRpQCT, which are not readily accessible in Africa, and open the door to better targeting and treatment of bone disease in people with DM residing in Africa.

### 4.1. Strengths and limitations

This systematic review and meta-analysis investigated the prevalence of previous fractures and fracture risk in adults of African ancestry with DM. These results have significant implications for clinical practice and public health, especially given the under-recognition of skeletal complications in DM in people of Africa ancestry. Clinicians should be attuned to addressing the risk factors for fractures (i.e. falls prevention) in Black adults with DM. Additionally, policymakers may need to consider broader health measures to reduce the morbidity and mortality associated with fractures in DM, such as improved screening or changes to subsidisation of treatment. Regardless, there are several important limitations to consider. Firstly, the paucity of studies in this review may have overestimated the effect sizes of our primary outcomes and limits the generalisability of our results. We were also unable to find fracture data in Black adults with DM living in Africa, although this may be due to the English language restriction in our search strategy. Consequently, there is an urgent need for further studies to better characterise the epidemiology of fractures in Black adults with DM, especially among those living in Africa. Moreover, we were unable to differentiate the contribution of T1DM and T2DM to fracture risk given the small number of studies. Even so, it is estimated that T2DM constitutes approximately 90 % of all DM cases [65]. Thus, it is likely that the majority of data in studies with unspecified DM pertain to people with T2DM [13]. Finally, we did not examine the impact of BMD as this was not accounted for in many studies. However, other bone properties may play a more important role in fracture risk given the higher BMD observed in T2DM [60].

## 5. Conclusion

In conclusion, adults of African ancestry with DM have an increased risk of incident fractures compared to those without DM. The burden of bone health in DM is further compounded by the relatively high prevalence of previous fractures in this population. With a rapidly increasing rate of both DM and fragility fractures in Africa and the exponential rise in population in this continent by 2050, improved and cost-sensitive diagnostics and therapeutic approaches are required to enable early identification and targeted treatment of individuals at higher risk of fractures.

## Supplementary Material

Supplemental Material

## Figures and Tables

**Table 1:** Characteristics of studies reporting prevalence of previous fractures in Black adults with DM.

						Black adults with DM				White adults with DM			
		
Author (year)	Cohort	DM type	Age at enrolment (y)	Sex	Fracture definition	Sex (% f)	Fracture (n)	DM (n)	Prevalence (%)	Sex (% f)	Fracture (n)	DM (n)	Prevalence (%)

Cauley, 2005^b^ [36], USA	SOF	N/A	≥ 65	Women only	Previous fractures > age 50 years	100	21	112	18.8	100	173	487	35.5
Hill, 2008^c^ [29], Trinidad and Tobago	The Tobago Bone Health Study	N/A	40–93	Men only	Previous fractures ^a^	0	89	472	18.9	N/A	N/A	N/A	N/A
Strotmeyer, 2004^b^ [33], USA	Health ABC	T2DM	70–79	Mixed	Previous fractures > age 45 years	53.4	51	324	15.7	28.9	48	242	19.8
Yu, 2014^c^ [31], USA	SWAN	T2DM	56–66	Women only	Previous fracture > age 20 (excluding hand, foot, and face)	100	4	22	18.2	N/A	N/A	N/A	N/A
Napoli, 2014^b^ [34], USA	MrOS	Both	≥ 65	Men only	Previous fractures ≥ age 50 years	0	10	63	15.9	0	163	738	22.1
Lenchik, 2018^b^ [30], USA	AA-DHS	T2DM	> 30	Mixed	Prevalent vertebral fractures	57.9	74	675	11.0	N/A	N/A	N/A	N/A
Cauley, 2008^c^ [32], USA	SOF	N/A	≥ 65	Women only	Prevalent vertebral fractures	100	12	80	15.0	100	83	515	16.1

AA-DHS = African American-Diabetes Heart Study; Health ABC = Health, Aging, and Body Composition Study; MrOS = Fracture in Men Study; SOF = Study of Osteoporotic Fractures; SWAN = Study of Women's Health Across the Nation

Abbreviation: N/A = not available; T2DM = type 2 diabetes mellitus

a Minimum age from when previous fracture were recorded not specified Study design:

b Prospective

c Cross-sectional

**Table 2:** Characteristics of studies reporting incident fracture risk in Black adults with DM.

								Black adults with DM			White adults with DM		
		
Author (year)	Cohort name	DM type	Fol-up y (SD)	Age at enrolment(y)	Sex	Fracture site	Measure of fracture risk	Diabetes (n)	Sex (% f)	Risk	Diabetes (n)	Sex (% f)	Risk

Schafer, 2010^b^ [38], USA	Health ABC	N/A	8.2 (2.3)	70–79	Mixed	Non-vertebral fractures	HR	340	52.4	1.67 (1.03, 2.71)^d^	318	37.1	1.29 (0.91, 1.85)^d^
Looker, 2015^c^ [18], USA	NHANESIII and NHANES1999–2004	Both	6.7	≥ 65	Mixed	Any non-skull fractures	HR	178	63.5	1.87 (1.02, 3.40)^e^	398	55.3	1.22 (0.93, 1.61)^e^
Cauley, 2007^b^ [35], USA	WHI	N/A	8.0 (2.6)	50–79	Women only	All fractures except fingers, toes, face, skull, or sternum	HR	1774	100	1.36 (1.15, 1.60)^f^	4453	100	1.36 (1.27, 1.45)^f^
Schneider, 2013^b^ [37], USA	ARIC	T2DM	NS ^a^	45–64	Mixed	Any incident fracture hospitalization	HR	571	65.3	1.92 (1.33, 2.77)^g^	624	51.4	1.68 (1.32, 2.14)^g^
Napoli, 2014^b^ [34], USA	MrOS	Both	9.1 (2.7)	≥ 65	Men only	Non-vertebral fractures	HR	63	0	6.94 (1.49, 32.2)^h^	738	0	1.05 (0.85, 1.29)^h^
Bonds, 2006^b^ [19], USA	WHI-OS	T2DM	7	50–79	Women only	Vertebral, shoulder, upper arm, lower arm, wrist, hip, upper leg, lower leg, and foot	RR	1091	100	1.33 (1.00, 1.75)^i^	3443	100	1.18 (1.08, 1.29)^i^
Strotmeyer, 2005^b^ [20], USA	Health ABC	T2DM	4.5 (1.1)	70–79	Mixed	Any fractures	RR	324	53.4	1.87 (1.11, 3.17)^j^	1730	51.4	1.39 (0.98, 1.96)^j^

ARIC = Atherosclerosis Risk in Communities Study; Health ABC = Health, Aging, and Body Composition Study; MrOS = The Osteoporotic Fracture in Men Study; NHANES = National Health and Nutrition Examination Survey; WHI = Women's Health Initiative

Abbreviation: HR = Hazard ratio; N/A = not available; T2DM = type 2 diabetes mellitus; RR = Relative risk

a Median of 20 years of follow-up Study design:

b Prospective

c Retrospective Controlled variables:

d Age, sex, clinic site, and total hip BMD

e Age, sex, survey, BMI, self-rated physical activity compared to others, hospital visits in past year, and smoking

f Age

g Age and sex

h Age, race, clinic site, total hip BMD, number of falls in previous year, BMI, history of fracture age 50+, history of stroke, history of heart attack, eGFR, depression, tricyclic antidepressant use, current smoker, grip strength, uses arms to stand up, hours sitting upright during day

i Age, weight, height, time-dependent history of falls, previous fracture, history of osteoporosis, trouble seeing at baseline, alcohol or tobacco use, calcium and vitamin D intake, exercise, bisphosphonate, estrogen, steroid, insulin, SERM, or thyroid hormone use

j Sex, age, site, hip BMD, LM, FM, abdominal visceral fat.

## Data Availability

The authors do not have permission to share data.

## Appendix A. Supplementary data

Supplementary data to this article can be found online at https://doi.org/10.1016/j.bone.2024.117133.
