# Supplementary material for: Epidemiology of fractures in adults of African ancestry with diabetes mellitus: A systematic review and meta-analysis
Source: Bone. Author manuscript; Available in PMC 2026 Apr 6. (PMC13051307; doi:10.1016/j.bone.2024.117133)
Supplement: Supplemental Material [file NIHMS2156582-supplement-Supplemental_Material.docx]

**Supplementary Table 1: Search strategies for systematic review**

Ovid MEDLINE(R) ALL <1946 to November 22, 2023>

1 (Africa or Africa* or black* or Algeria or Angola or Benin or Botswana or Burkina Faso or Burundi or Cameroon or Canary Islands or Cape Verde or Central African Republic or Chad or Comoros or Congo or Democratic Republic of Congo or Djibouti or Egypt or Equatorial Guinea or Eritrea or Ethiopia or Gabon or Gambia or Ghana or Guinea or Guinea Bissau or Ivory Coast or Cote d'Ivoire or Jamahiriya or Jamahiryia or Kenya or Lesotho or Liberia or Libya or Libia or Madagascar or Malawi or Mali or Mauritania or Mauritius or Mayote or Morocco or Mozambique or Mocambique or Namibia or Niger or Nigeria or Principe or Reunion or Rwanda or Sao Tome or Senegal or Seychelles or Sierra Leone or Somalia or South Africa or St Helena or Sudan or Swaziland or Tanzania or Togo or Tunisia or Uganda or Western Sahara or Zaire or Zambia or Zimbabwe or Central Africa or Central African or West Africa or West African or Western Africa or Western African or East Africa or East African or Eastern Africa or Eastern African or North Africa or North African or Northern Africa or Northern African or South African or Southern Africa or Southern African or subSaharan Africa or subSaharan African or subSaharan Africa or subSaharan African).mp. [mp=title, book title, abstract, original title, name of substance word, subject heading word, floating sub-heading word, keyword heading word, organism supplementary concept word, protocol supplementary concept word, rare disease supplementary concept word, unique identifier, synonyms, population supplementary concept word, anatomy supplementary concept word] 961318

2 exp Africa/ 330180

3 1 or 2 961513

4 exp Osteoporosis, Postmenopausal/ or exp Osteoporosis/ 63608

5 exp Fractures, Bone/ 211050

6 (fracture* or osteoporo* or fragil*).mp. [mp=title, book title, abstract, original title, name of substance word, subject heading word, floating sub-heading word, keyword heading word, organism supplementary concept word, protocol supplementary concept word, rare disease supplementary concept word, unique identifier, synonyms, population supplementary concept word, anatomy supplementary concept word] 480643

7 4 or 5 or 6 483057

8 diabet*.mp. [mp=title, book title, abstract, original title, name of substance word, subject heading word, floating sub-heading word, keyword heading word, organism supplementary concept word, protocol supplementary concept word, rare disease supplementary concept word, unique identifier, synonyms, population supplementary concept word, anatomy supplementary concept word] 860019

9 3 and 7 and 8 230

*******************************

Embase Classic+Embase <1947 to 2023 November 22>

1 (Africa or Africa* or black* or Algeria or Angola or Benin or Botswana or Burkina Faso or Burundi or Cameroon or Canary Islands or Cape Verde or Central African Republic or Chad or Comoros or Congo or Democratic Republic of Congo or Djibouti or Egypt or Equatorial Guinea or Eritrea or Ethiopia or Gabon or Gambia or Ghana or Guinea or Guinea Bissau or Ivory Coast or Cote d'Ivoire or Jamahiriya or Jamahiryia or Kenya or Lesotho or Liberia or Libya or Libia or Madagascar or Malawi or Mali or Mauritania or Mauritius or Mayote or Morocco or Mozambique or Mocambique or Namibia or Niger or Nigeria or Principe or Reunion or Rwanda or Sao Tome or Senegal or Seychelles or Sierra Leone or Somalia or South Africa or St Helena or Sudan or Swaziland or Tanzania or Togo or Tunisia or Uganda or Western Sahara or Zaire or Zambia or Zimbabwe or Central Africa or Central African or West Africa or West African or Western Africa or Western African or East Africa or East African or Eastern Africa or Eastern African or North Africa or North African or Northern Africa or Northern African or South African or Southern Africa or Southern African or subSaharan Africa or subSaharan African or subSaharan Africa or subSaharan African).mp. [mp=title, abstract, heading word, drug trade name, original title, device manufacturer, drug manufacturer, device trade name, keyword heading word, floating subheading word, candidate term word] 1328167

2 exp Africa/ 442540

3 1 or 2 1328404

4 exp Osteoporosis, Postmenopausal/ or exp Osteoporosis/ 161921

5 exp Fractures, Bone/ 399910

6 (fracture* or osteoporo* or fragil*).mp. [mp=title, abstract, heading word, drug trade name, original title, device manufacturer, drug manufacturer, device trade name, keyword heading word, floating subheading word, candidate term word] 705827

7 4 or 5 or 6 716280

8 diabet*.mp. [mp=title, abstract, heading word, drug trade name, original title, device manufacturer, drug manufacturer, device trade name, keyword heading word, floating subheading word, candidate term word] 1534919

9 3 and 7 and 8 1043

*******************************

CINAHL Complete

| **#** | **Query** | **Limiters/Expanders** | **Last Run Via** | **Results** |
| --- | --- | --- | --- | --- |
| S1 | ( Africa* or black* or Algeria or Angola or Benin or Botswana or "Burkina Faso" or Burundi or Cameroon or "Canary Islands" or "Cape Verde" or "Central African Republic" or Chad or Comoros or Congo or "Democratic Republic of Congo" or Djibouti or Egypt or "Equatorial Guinea" or Eritrea or Ethiopia or Gabon or Gambia or Ghana or Guinea or "Guinea Bissau" or "Ivory Coast" or "Cote d'Ivoire" or Jamahiriya or Jamahiryia or Kenya or Lesotho or Liberia or Libya or Libia or Madagascar or Malawi or Mali or Mauritania or Mauritius or Mayote or Morocco or Mozambique or Mocambique or Namibia or Niger or Nigeria or Principe or Reunion or Rwanda or "Sao Tome" or Senegal or Seychelles or "Sierra Leone" or Somalia or "South Africa" or "St Helena" or Sudan or Swaziland or Tanzania or Togo or Tunisia or Uganda or "Western Sahara" or Zaire or Zambia or Zimbabwe or "Central Africa" or "Central African" or "West Africa" or "West African" or "Western Africa" or "Western African" or "East Africa" or "East African" or "Eastern Africa" or "Eastern African" or "North Africa" or "North African" or "Northern Africa" or "Northern African" or "South African" or "Southern Africa" or "Southern African" or "subSaharan Africa" or "subSaharan African" ) AND ( fracture* or osteoporo* or fragil* ) AND diabet* | Search modes - Boolean/Phrase | Interface - EBSCOhost Research Databases  Search Screen - Advanced Search  Database - CINAHL Complete | 107 |

*******************************

SCOPUS

( TITLE-ABS-KEY ( africa* OR black* OR algeria OR angola OR benin OR botswana OR "burkina faso" OR burundi OR cameroon OR "canary islands" OR "cape verde" OR "central african republic" OR chad OR comoros OR congo OR "democratic republic of congo" OR djibouti OR egypt OR "equatorial guinea" OR eritrea OR ethiopia OR gabon OR gambia OR ghana OR guinea OR "guinea bissau" OR "ivory coast" OR "cote d'ivoire" OR jamahiriya OR jamahiryia OR kenya OR lesotho OR liberia OR libya OR libia OR madagascar OR malawi OR mali OR mauritania OR mauritius OR mayote OR morocco OR mozambique OR mocambique OR namibia OR niger OR nigeria OR principe OR reunion OR rwanda OR "sao tome" OR senegal OR seychelles OR "sierra leone" OR somalia OR "south africa" OR "st helena" OR sudan OR swaziland OR tanzania OR togo OR tunisia OR uganda OR "western sahara" OR zaire OR zambia OR zimbabwe OR "central africa" OR "central african" OR "west africa" OR "west african" OR "western africa" OR "western african" OR "east africa" OR "east african" OR "eastern africa" OR "eastern african" OR "north africa" OR "north african" OR "northern africa" OR "northern african" OR "south african" OR "southern africa" OR "southern african" OR "subsaharan africa" OR "subsaharan african" ) AND TITLE-ABS-KEY ( fracture* OR osteoporo* OR fragil* ) AND TITLE-ABS-KEY ( diabet* ) )

788 results

*******************************

Google Scholar and African Journals Online

“fracture”, “diabetes”, “risk”, “prevalence”, “black”

**Supplementary Table 2a: DM status and history of fractures > 50 among African American (AA) and White men in the Osteoporotic Fracture in Men (MrOS) Study**

|  | AA men with no diabetes  (n=181) | AA men with diabetes  (n=63) | White men with diabetes  (n=738) |
| --- | --- | --- | --- |
| Any fracture > 50 years of age | 37 (20.4) | 10 (15.9) | 163 (22.1) |

**Supplementary Table 2b: Adjusted hazard ratio (HR) for fracture by diabetes status in older African American (AA) and White men with diabetes mellitus in the Osteoporotic Fracture in Men (MrOS) Study**

| Model |  | HR (95% CI) in AA men ^a^ | HR (95% CI) in White men ^b^ |
| --- | --- | --- | --- |
|  |  |  |  |
| 1. Adjusted for age, race, clinic |  | 1.87 (0.61, 5.79) |  |
| 2. Adjusted for Model 1 plus total hip BMD |  | 2.84 (0.89, 9.04) |  |
| 3. Adjusted for Model 1 plus falls in the year before baseline |  | 1.56 (0.47, 5.17) |  |
| 4. Multivariable model ^c^ |  | 6.94 (1.49, 32.2) | 1.05 (0.85, 1.29) |

^a^ Reference group is AA men without diabetes

^b^ Reference group is White men without diabetes

^c^ Adjusted for age, race, clinic site, total hip BMD, number of falls in previous year, BMI, history of fracture age 50+, history of stroke, history of heart attack, eGFR, depression, tricyclic antidepressant use, current smoker, grip strength, uses arms to stand up, hours sitting upright during day.

**Supplementary Table 2c: Prevalence of previous fractures among African Caribbean men (n=2286) with history of diabetes and non-missing fracture from Visit 3 in The Tobago Bone Health Study**

|  | History of diabetes  (n=572) | No history of diabetes  (n=1814) |
| --- | --- | --- |
| Previous fracture  n (%) | 89 (18.9) | 372 (20.5) |

**Supplementary Table 2d: DM status and fracture since age 50 y among African American (AA) and White women in the Study of Osteoporotic Fractures (SOF)**

|  | AA women with diabetes  (n=112) | White women with diabetes  (n=487) |
| --- | --- | --- |
| Fracture since age 50 y, n (%) | 21 (19.1) | 173 (35.9) |

**Supplementary Table 2e: Adjusted hazard ratio (HR) for incident clinical fractures by diabetes status in Black and White adults with diabetes mellitus (DM) in the The Health, Aging, and Body Composition (Health ABC) Study**

| Model |  | Black adults with DM, incident clinical fracture ^a^ | White adults with DM, incident clinical fracture ^b^ |
| --- | --- | --- | --- |
|  |  | HR (95% CI) | HR (95% CI) |
|  |  |  |  |
| 1. Adjusted for age |  | 1.06 (0.98, 1.15) |  |
| 2. Adjusted for sex |  | 1.27 (0.68, 2.37) |  |
| 3. Adjusted for site |  | 1.58 (0.97, 2.57) |  |
| 4. Adjusted for age, sex, clinic site, and total hip BMD |  | 1.67 (1.03, 2.71) | 1.29 (0.91, 1.85) |

^a^ Reference group is Black adults without diabetes.

^b^ Reference group is White adults without diabetes.

**Supplementary Table 2f: Adjusted hazard ratio (HR) for incident fracture-related hospitalisation by diabetes status in Black and White adults with DM in the Atherosclerosis Risk in Communities (ARIC) Study**

| Model | Black adults with DM, HR (95% CI) ^a^ | White adults with DM, HR (95% CI) ^b^ |
| --- | --- | --- |
|  |  |  |
| 1. Adjusted for age | 1.19 (1.05, 1.35) | 1.46 (1.37, 1.55) |
| 2. Adjusted for sex | 0.81 (0.60, 1.09) | 1.43 (1.25, 1.64) |
| 3. Adjusted for age and sex | 1.92 (1.33, 2.77) | 1.68 (1.32, 2.14) |

^a^ Reference group is Black adults without diabetes.

^b^ Reference group is White adults without diabetes.

**Supplementary Table 2g: Adjusted relative risk (RR) for incident clinical fractures in Black and White adults with diabetes mellitus (DM)** **in** **The Health, Aging, and Body Composition (Health ABC) Study**

| Adjustments for Covariates | RR (95% CI) in Black adults with DM ^a^ | RR (95% CI) in White adults with DM ^b^ |
| --- | --- | --- |
|  |  |  |
| Sex, race, age site | 1.34 (0.85, 2.13) | 1.21 (0.86, 1.69) |
| and hip BMD | 1.77 (1.11, 2.83) | 1.39 (0.99, 1.95) |
| and LM, FM, ab visceral fat | 1.73 (1.03, 2.90) | 1.28 (0.90, 1.83) |
| and hip BMD, LM, FM, ab visceral fat | 1.87 (1.11, 3.17) | 1.39 (0.98, 1.96) |

^a^ Reference group is Black adults without diabetes.

^b^ Reference group is White adults without diabetes.

**Supplementary Table 3:** **Exclusion of full text studies with reasons**

| **Reason for exclusion** | **Studies excluded on this basis** |
| --- | --- |
| No fracture outcomes reported | 35   1. Schwartz AV, Sellmeyer DE, Strotmeyer ES, Tylavsky FA, Feingold KR, Resnick HE, Shorr RI, Nevitt MC, Black DM, Cauley JA, Cummings SR. Diabetes and bone loss at the hip in older black and white adults. Journal of Bone and Mineral Research. 2005 Apr;20(4):596-603. 2. Akeroyd JM, Suarez EA, Bartali B, Chiu GR, Yang M, Schwartz AV, Araujo AB. Differences in skeletal and non-skeletal factors in a diverse sample of men with and without type 2 diabetes mellitus. Journal of diabetes and its complications. 2014 Sep 1;28(5):679-83. 3. Noel SE, Mangano KM, Griffith JL, Wright NC, Dawson‐Hughes B, Tucker KL. Prevalence of osteoporosis and low bone mass among Puerto Rican older adults. Journal of Bone and Mineral Research. 2018 Mar;33(3):396-403. 4. Chode S, Malmstrom TK, Miller DK, Morley JE. Frailty, diabetes, and mortality in middle-aged African Americans. The journal of nutrition, health & aging. 2016 Oct;20:854-9. 5. Negash W, Assefa T, Sahiledengle B, Tahir A, Regassa Z, Feleke Z, Regasa T, Tekalegn Y, Mamo A, Teferu Z, Solomon D. Prevalences of diabetic foot ulcer and foot self-care practice, and associated factors in adult patients with diabetes in south-east Ethiopia. Journal of International Medical Research. 2022 Oct;50(10):03000605221129028. 6. Cauley JA, Karlamangla AS, Ruppert K, Lian Y, Huang M, Harlow S, Finkelstein JS, Greendale GA. Race/ethnic difference in trabecular bone score in midlife women: The Study of Women’s Health Across the Nation (SWAN). Archives of osteoporosis. 2021 Dec;16(1):91. 7. Leone M, Ciccacci F, Orlando S, Petrolati S, Guidotti G, Majid NA, Tolno VT, Sagno J, Thole D, Corsi FM, Bartolo M. Pandemics and burden of stroke and epilepsy in sub-Saharan Africa: experience from a longstanding health programme. International Journal of Environmental Research and Public Health. 2021 Mar 9;18(5):2766. 8. Ruppert K, Cauley J, Lian Y, Zgibor JC, Derby C, Solomon DH. The effect of insulin on bone mineral density among women with type 2 diabetes: a SWAN Pharmacoepidemiology study. Osteoporosis International. 2018 Feb;29:347-54. 9. Freedman BI, Divers J, Russell GB, Palmer ND, Wagenknecht LE, Smith SC, Xu J, Carr JJ, Bowden DW, Register TC. Vitamin D associations with renal, bone, and cardiovascular phenotypes: African American-Diabetes Heart Study. The Journal of Clinical Endocrinology & Metabolism. 2015 Oct 1;100(10):3693-701. 10. Hanlon JT, Landerman LR, Fillenbaum GG, Studenski S. Falls in African American and white community-dwelling elderly residents. The Journals of Gerontology Series A: Biological Sciences and Medical Sciences. 2002 Jul 1;57(7):M473-8. 11. Schafer AL, Napoli N, Lui L, Schwartz AV, Black DM, Study of Osteoporotic Fractures. Serum 25‐hydroxyvitamin D concentration does not independently predict incident diabetes in older women. Diabetic medicine. 2014 May;31(5):564-9. 12. Harris SS. Vitamin D and african americans. The Journal of nutrition. 2006 Apr 1;136(4):1126-9. 13. Hicks CW, Wang D, Daya N, Juraschek SP, Matsushita K, Windham BG, Selvin E. The association of peripheral neuropathy detected by monofilament testing with risk of falls and fractures in older adults. Journal of the American Geriatrics Society. 2023 Mar 21. 14. Dawson-Hughes B, Bouxsein M, Shea K. Bone material strength in normoglycemic and hyperglycemic black and white older adults. Osteoporosis International. 2019 Dec;30:2429-35. 15. Divers J, Register TC, Langefeld CD, Wagenknecht LE, Bowden DW, Carr JJ, Hightower RC, Xu J, Hruska KA, Freedman BI. Relationships between calcified atherosclerotic plaque and bone mineral density in African Americans with type 2 diabetes. Journal of Bone and Mineral Research. 2011 Jul;26(7):1554-60. 16. Hansen R, Shibuya N, Jupiter DC. An updated epidemiology of foot and ankle fractures in the United States: complications, mechanisms, and risk factors. The Journal of Foot and Ankle Surgery. 2022 Sep 1;61(5):1034-8. 17. Strotmeyer ES, Cauley JA, Schwartz AV, de Rekeneire N, Resnick HE, Zmuda JM, Shorr RI, Tylavsky FA, Vinik AI, Harris TB, Newman AB. Reduced peripheral nerve function is related to lower hip BMD and calcaneal QUS in older white and black adults: the Health, Aging, and Body Composition Study. Journal of Bone and Mineral Research. 2006 Nov;21(11):1803-10. 18. Schwartz AV, Sellmeyer DE, Vittinghoff E, Palermo L, Lecka-Czernik B, Feingold KR, Strotmeyer ES, Resnick HE, Carbone L, Beamer BA, Park SW. Thiazolidinedione use and bone loss in older diabetic adults. The Journal of Clinical Endocrinology & Metabolism. 2006 Sep 1;91(9):3349-54. 19. Geng Y, Lo JC, Brickner L, Gordon NP. Racial-ethnic differences in fall prevalence among older women: a cross-sectional survey study. BMC geriatrics. 2017 Dec;17:1-7. 20. Kibachio JM, Omolo J, Muriuki Z, Juma R, Karugu L, Ng'ang'a Z. Risk factors for diabetic foot ulcers in type 2 diabetes: a case control study, Nyeri, Kenya. African Journal of Diabetes Medicine. 2013 May 1;21(1). 21. McGrath RP, Al Snih S, Markides KS, Faul JD, Vincent BM, Hall OT, Peterson MD. The burden of health conditions across race and ethnicity for aging Americans: Disability-adjusted life years. Medicine. 2019 Nov;98(46). 22. Hsu S, Criqui MH, Ginsberg C, Hoofnagle AN, Ix JH, McClelland RL, Michos ED, Shea SJ, Siscovick D, Zelnick LR, Kestenbaum BR. Biomarkers of Vitamin D Metabolism and Hip and Vertebral Fracture Risk: The Multi‐Ethnic Study of Atherosclerosis. JBMR plus. 2022 Dec;6(12):e10697. 23. Huang JF, Wu QN, Zheng XQ, Sun XL, Wu CY, Wang XB, Wu CW, Wang B, Wang XY, Bergman M, Wu AM. The characteristics and mortality of osteoporosis, osteomyelitis, or rheumatoid arthritis in the diabetes population: a Retrospective Study. International Journal of Endocrinology. 2020 Nov 7;2020:1-3. 24. Simske NM, Benedick A, Audet MA, Vallier HA. Ankle fractures in patients over age 55 years: Predictors of functional outcome. OTA International. 2020 Sep;3(3). 25. Paruk F, Matthews G, Gregson CL, Cassim B. Hip fractures in South Africa: mortality outcomes over 12 months post-fracture. Archives of Osteoporosis. 2020 Dec;15:1-0. 26. Becker C, Crow S, Toman J, Lipton C, McMahon DJ, Macaulay W, Siris E. Characteristics of elderly patients admitted to an urban tertiary care hospital with osteoporotic fractures: correlations with risk factors, fracture type, gender and ethnicity. Osteoporosis International. 2006 Mar;17:410-6. 27. Madduri S, Shenoy SS, Nunlee-Bland GL, Cherqaoui R, Bunton-Young C, Rampaul M, Odonkor WA, Ganta VA, Duerinckx AJ, Hasan N, Archer JA. Bone mineral density and fractures in urban African Americans with type 2 diabetes. Journal of Clinical Densitometry. 2014;3(17):424. 28. Hanlon JT, Landerman LR, Fillenbaum GG, Studenski S. Falls in African American and white community-dwelling elderly residents. The Journals of Gerontology Series A: Biological Sciences and Medical Sciences. 2002 Jul 1;57(7):M473-8. 29. Margolis KL, Palermo L, Vittinghoff E, Evans GW, Atkinson HH, Hamilton BP, Josse RG, O’Connor PJ, Simmons DL, Tiktin M, Schwartz AV. Intensive blood pressure control, falls, and fractures in patients with type 2 diabetes: the ACCORD trial. Journal of general internal medicine. 2014 Dec;29:1599-606. 30. Dela SS, Paruk F, Cassim B. Clinical profile, risk factors and functional outcomes in women and men presenting with hip fractures in KwaZulu-Natal, South Africa. Archives of osteoporosis. 2022 Dec 9;18(1):7. 31. Baidoo PK, Odei JB, Ansu V, Segbefia M, Holdbrook-Smith H. Predictors of hip fracture mortality in Ghana: a single-center prospective study. Archives of Osteoporosis. 2021 Dec;16:1-8. 32. Adrosy TI, Hanafy MI, Hamoud HS, Negm AA. AB1062 Patterns of musculoskeletal system involvement in patients with type i and type ii diabetes mellitus. 33. Chode S, Malmstrom TK, Miller DK, Morley JE. Frailty, diabetes, and mortality in middle-aged African Americans. The journal of nutrition, health & aging. 2016 Oct;20:854-9. 34. Figaro MK, Long DM, May ME, Ndetan H, Cook A, Conway RB. Racial Variation in the Relationship of Glycemic Control with Fracture Risk in Elderly Patients with Diabetes. Diabetes, Metabolic Syndrome and Obesity. 2020 Nov 5:4153-5. 35. Kidambi S, Partington S, Binkley N. Low bone mass prevalence and osteoporosis risk factor assessment in African American Wisconsin women. Wisconsin Medical Journal. 2005 Nov 1;104(8):59-60. |
| Fracture outcomes not reported in Black participants with diabetes | 23   1. Taylor AJ, Gary LC, Arora T, Becker DJ, Curtis JR, Kilgore ML, Morrisey MA, Saag KG, Matthews R, Yun H, Smith W. Clinical and demographic factors associated with fractures among older Americans. Osteoporosis international. 2011 Apr;22:1263-74. 2. Strotmeyer ES, Kamineni A, Cauley JA, Robbins JA, Fried LF, Siscovick DS, Harris TB, Newman AB. Potential explanatory factors for higher incident hip fracture risk in older diabetic adults. Current Gerontology and Geriatrics Research. 2011 Jan 1;2011. 3. Taher Y, Ben Emhemed HM, Tawati AM. The menopausal experience of Libyan women. JMJ. 2009;9(3):184-90. 4. Khalil N, Sutton-Tyrrell K, Strotmeyer ES, Greendale GA, Vuga M, Selzer F, Crandall CJ, Cauley JA. Menopausal bone changes and incident fractures in diabetic women: a cohort study. Osteoporosis international. 2011 May;22:1367-76. 5. Schwartz AV, Garnero P, Hillier TA, Sellmeyer DE, Strotmeyer ES, Feingold KR, Resnick HE, Tylavsky FA, Black DM, Cummings SR, Harris TB. Pentosidine and increased fracture risk in older adults with type 2 diabetes. The Journal of Clinical Endocrinology & Metabolism. 2009 Jul 1;94(7):2380-6. 6. Schwartz AV, Vittinghoff E, Bauer DC, Hillier TA, Strotmeyer ES, Ensrud KE, Donaldson MG, Cauley JA, Harris TB, Koster A, Womack CR. Association of BMD and FRAX score with risk of fracture in older adults with type 2 diabetes. Jama. 2011 Jun 1;305(21):2184-92. 7. Huynh HL, Fan L, Germosen C, Bucovsky M, Colon I, Kil N, Agarwal S, Walker M. Thiazide use and skeletal microstructure: Results from a multi-ethnic study. Bone Reports. 2022 Jun 1;16:101589. 8. Wolinsky FD, Bentler SE, Liu L, Obrizan M, Cook EA, Wright KB, Geweke JF, Chrischilles EA, Pavlik CE, Ohsfeldt RL, Jones MP. Recent hospitalization and the risk of hip fracture among older Americans. Journals of Gerontology Series A: Biomedical Sciences and Medical Sciences. 2009 Feb 1;64(2):249-55. 9. Stehman-Breen CO, Sherrard DJ, Alem AM, Gillen DL, Heckbert SR, Wong CS, Ball A, Weiss NS. Risk factors for hip fracture among patients with end-stage renal disease. Kidney international. 2000 Nov 1;58(5):2200-5.Americans. Journals of Gerontology Series A: Biomedical Sciences and Medical Sciences. 2009 Feb 1;64(2):249-55. 10. Sheu Y, Bunker CH, Jonnalagadda P, Cvejkus RK, Patrick AL, Wheeler VW, Gordon CL, Zmuda JM. Rates of and risk factors for trabecular and cortical BMD loss in middle‐aged and elderly African‐ancestry men. Journal of Bone and Mineral Research. 2015 Mar;30(3):543-53. 11. Sutton SS, Magagnoli J, Cummings TH, Hardin JW, Edun B, Beaubrun A. Chronic kidney disease, cardiovascular disease, and osteoporotic fractures in patients with and without HIV in the US Veteran’s Affairs Administration System. Current Medical Research and Opinion. 2019 Jan 2;35(1):117-25. 12. Putman MS, Yu EW, Lin D, Darakananda K, Finkelstein JS, Bouxsein ML. Differences in trabecular microstructure between black and white women assessed by individual trabecular segmentation analysis of HR‐pQCT images. Journal of Bone and Mineral Research. 2017 May;32(5):1100-8. 13. Kuipers AL, Egwuogu H, Evans RW, Patrick AL, Youk A, Bunker CH, Zmuda JM. Renal function and bone loss in a cohort of Afro‐Caribbean men. Journal of Bone and Mineral Research. 2015 Dec;30(12):2215-20. 14. Al Snih S, Kaushik V, Eschbach K, Markides K. Ethnic differences in physical performance in older Americans: data from the Third National Health and Nutrition Examination Survey (1988–1994). Aging clinical and experimental research. 2008 Apr;20:139-44. 15. Chen Z, Thomson CA, Aickin M, Nicholas JS, Van Wyck D, Lewis CE, Cauley JA, Bassford T, Short list of Women's Health Initiative Investigators. The relationship between incidence of fractures and anemia in older multiethnic women. Journal of the American Geriatrics Society. 2010 Dec;58(12):2337-44. 16. Sheu Y, Cauley JA, Bunker CH, Wheeler VW, Patrick AL, Gordon CL, Kammerer CM, Zmuda JM. Correlates of trabecular and cortical volumetric BMD in men of African ancestry. Journal of Bone and Mineral Research. 2009 Dec;24(12):1960-8. 17. LaCroix AZ, Rillamas-Sun E, Buchner D, Evenson KR, Di C, Lee IM, Marshall S, LaMonte MJ, Hunt J, Tinker LF, Stefanick M. The objective physical activity and cardiovascular disease health in older women (OPACH) study. BMC public health. 2017 Dec;17(1):1-2. 18. Cauley JA, Fullman RL, Stone KL, Zmuda JM, Bauer DC, Barrett-Connor E, Ensrud K, Lau EM, Orwoll ES, Mr. OS Research Group. Factors associated with the lumbar spine and proximal femur bone mineral density in older men. Osteoporosis International. 2005 Dec;16:1525-37. 19. Cauley JA, Lui LY, Ensrud KE, Zmuda JM, Stone KL, Hochberg MC, Cummings SR. Bone mineral density and the risk of incident nonspinal fractures in black and white women. Jama. 2005 May 4;293(17):2102-8. 20. Crandall CJ, Larson J, Wright NC, Laddu D, Stefanick ML, Kaunitz AM, Watts NB, Wactawski-Wende J, Womack CR, Johnson KC, Carbone LD. Serial bone density measurement and incident fracture risk discrimination in postmenopausal women. JAMA internal medicine. 2020 Sep 1;180(9):1232-40. 21. Wallace LS, Ballard JE, Holiday DB, Wells HE. Comparison between 60 matched pairs of postmenopausal black and white women: analysis of risk factors related to bone mineral density. Maturitas. 2005 Nov 1;52(3-4):356-63. 22. Sheu Y, Cauley JA, Wheeler VW, Patrick AL, Bunker CH, Ensrud KE, Orwoll ES, Zmuda JM, Osteoporotic Fracture in Men (MrOS) Research Group. Age-related decline in bone density among ethnically diverse older men. Osteoporosis international. 2011 Feb;22:599-605. 23. Nelson DA, Beck TJ, Wu G, Lewis CE, Bassford T, Cauley JA, LeBoff MS, Going SB, Chen Z. Ethnic differences in femur geometry in the women's health initiative observational study. Osteoporosis international. 2011 May;22:1377-88. |
| No Black participants included in study | 6   1. Kilpadi KL, Eldabaje R, Schmitz JE, Ehler B, Thames TA, Joshi AP, Simmons III JW, Michalek JE, Fajardo RJ. Type 2 diabetes is associated with vertebral fractures in a sample of clinic-and hospital-based Latinos. Journal of immigrant and minority health. 2014 Jun;16:440-9. 2. Schwartz AV, Ewing SK, Porzig AM, McCulloch CE, Resnick HE, Hillier TA, Ensrud KE, Black DM, Nevitt MC, Cummings SR, Sellmeyer DE. Diabetes and change in bone mineral density at the hip, calcaneus, spine, and radius in older women. Frontiers in endocrinology. 2013 May 30;4:62. 3. Kanazawa I, Yano S, Yamaguchi T, Notsu Y, Nabika T, Sugimoto T. Relationships between dimethylarginine and the presence of vertebral fractures in type 2 diabetes mellitus. Clinical endocrinology. 2010 Oct;73(4):463-8. 4. Appiah D, Winters SJ, Muldoon SB, Hornung CA, Cauley JA. Androgens, bilateral oophorectomy, and cardiovascular disease mortality in postmenopausal women with and without diabetes: the study of osteoporotic fractures. Diabetes Care. 2015 Dec 1;38(12):2301-7. 5. Ensrud KE, Thompson DE, Cauley JA, Nevitt MC, Kado DM, Hochberg MC, Santora AC, Black DM. Prevalent vertebral deformities predict mortality and hospitalization in older women with low bone mass. Journal of the American geriatrics society. 2000 Mar;48(3):241-9. 6. Napoli N, Schwartz AV, Palermo L, Jin JJ, Wustrack R, Cauley JA, Ensrud KE, Kelly M, Black DM. Risk factors for subtrochanteric and diaphyseal fractures: the study of osteoporotic fractures. The Journal of Clinical Endocrinology & Metabolism. 2013 Feb 1;98(2):659-67. |
| Not appropriate control group | 2   1. Jain RK, Weiner MG, Zhao H, Williams KJ, Vokes T. Diabetes-related fracture risk is different in African Americans compared with Hispanics and Caucasians. The Journal of Clinical Endocrinology & Metabolism. 2019 Nov;104(11):5729-36. 2. Jain RK, Weiner MG, Zhao H, Vokes T. Comorbid conditions and GFR predict nonvertebral fractures in patients with diabetes in an ethnic-specific manner. The Journal of Clinical Endocrinology & Metabolism. 2020 Jun;105(6):e2168-75. |
| Participants with insulin resistance or impaired glucose tolerance | 2   1. Napoli N, Conte C, Pedone C, Strotmeyer ES, Barbour KE, Black DM, Samelson EJ, Schwartz AV. Effect of insulin resistance on BMD and fracture risk in older adults. The Journal of Clinical Endocrinology & Metabolism. 2019 Aug;104(8):3303-10. 2. Shieh A, Greendale GA, Cauley JA, Karvonen-Gutierrez CA, Karlamangla AS. Prediabetes and Fracture Risk Among Midlife Women in the Study of Women’s Health Across the Nation. JAMA Network Open. 2023 May 1;6(5):e2314835-. |
| Review articles | 2   1. Strotmeyer ES, Cauley JA. Diabetes mellitus, bone mineral density, and fracture risk. Current Opinion in Endocrinology, Diabetes and Obesity. 2007 Dec 1;14(6):429-35. 2. Schwartz AV. Epidemiology of fractures in type 2 diabetes. Bone. 2016 Jan 1;82:2-8. |

**Supplementary Table 4a:** **JBI (Joanna Briggs Institute) checklist for prevalence studies: scoring for studies included in systematic reviews/meta-analysis**

| JBI items^a^ | Cauley, 2005 | Hill, 2008 | Strotmeyer, 2004 | Yu, 2014 | Napoli, 2014 | Lenchik, 2018 | Cauley, 2008 |
| --- | --- | --- | --- | --- | --- | --- | --- |
| 1. Was the sample frame appropriate to address the target population? | Yes | Yes | Yes | Yes | Yes | Yes | Yes |
| 2. Were study participants sampled in an appropriate way? | No | Yes | Yes | Yes | No | No | No |
| 3. Was the sample size adequate? | Yes | Yes | Yes | No | No | Yes | No |
| 4. Were the study subjects and the setting described in detail? | Yes | Yes | Yes | Yes | Yes | Yes | Yes |
| 5. Was the data analysis conducted with sufficient coverage of the identified sample? | Yes | Yes | Yes | Yes | Yes | Yes | Yes |
| 6. Were valid methods used for the identification of the condition? | Yes | Yes | Yes | Yes | Yes | Yes | Yes |
| 7. Was the condition measured in a standard, reliable way for all participants? | Yes | Yes | Yes | Yes | Yes | Yes | Yes |
| 8. Was there appropriate statistical analysis? | Yes | Yes | Yes | Yes | Yes | Yes | Yes |
| 9. Was the response rate adequate, and if not, was the low response rate managed appropriately? | Yes | Unclear | Unclear | Yes | Unclear | Yes | No |
| Risk of bias | Low | Low | Low | Low | Moderate | Low | Moderate |

a. Low risk of bias: score > 70%

b. Moderate risk of bias: score between 50% and 69%

c. High risk of bias: score < 50%

**Supplementary Table 4b:** **JBI (Joanna Briggs Institute) checklist for cohort studies: scoring for studies included in systematic reviews/meta-analysis**

| JBI items | Schafer, 2010 | Looker, 2015 | Cauley, 2007 | Schneider, 2013 | Napoli, 2014 | Bonds, 2006 | Strotmeyer, 2005 |
| --- | --- | --- | --- | --- | --- | --- | --- |
| 1. Were the two groups similar and recruited from the same population? | Yes | Yes | Yes | Yes | Yes | Yes | Yes |
| 2. Were the exposures measured similarly to assign people to both exposed and unexposed groups? | Yes | Yes | Yes | Yes | Yes | Yes | Yes |
| 3. Was the exposure measured in a valid and reliable way? | Yes | Yes | Yes | Yes | Yes | Yes | Yes |
| 4. Were confounding factors identified? | Yes | Yes | Yes | Yes | Yes | Yes | Yes |
| 5. Were strategies to deal with confounding factors stated? | Yes | Yes | Yes | Yes | Yes | Yes | Yes |
| 6. Were the groups/participants free of the outcome at the start of the study (or at the moment of exposure)? | No | Yes | No | No | No | No | No |
| 7. Were the outcomes measured in a valid and reliable way? | Yes | Yes | Yes | Yes | Yes | Yes | Yes |
| 8. Was the follow up time reported and sufficient to be long enough for outcomes to occur? | Yes | Yes | Yes | No | Yes | Yes | Yes |
| 9. Was follow up complete, and if not, were the reasons to loss to follow up described and explored? | Unclear | Yes | Yes | Unclear | Unclear | Unclear | Unclear |
| 10. Were strategies to address incomplete follow up utilized? | Unclear | Yes | Unclear | Unclear | Unclear | Yes | Unclear |
| 11. Was appropriate statistical analysis used? | Yes | Yes | Yes | Yes | Yes | Yes | Yes |
| Risk of bias | Low | Low | Low | Moderate | Low | Low | Low |

a. Low risk of bias: score > 70%

b. Moderate risk of bias: score between 50% and 69%

c. High risk of bias: score < 50%


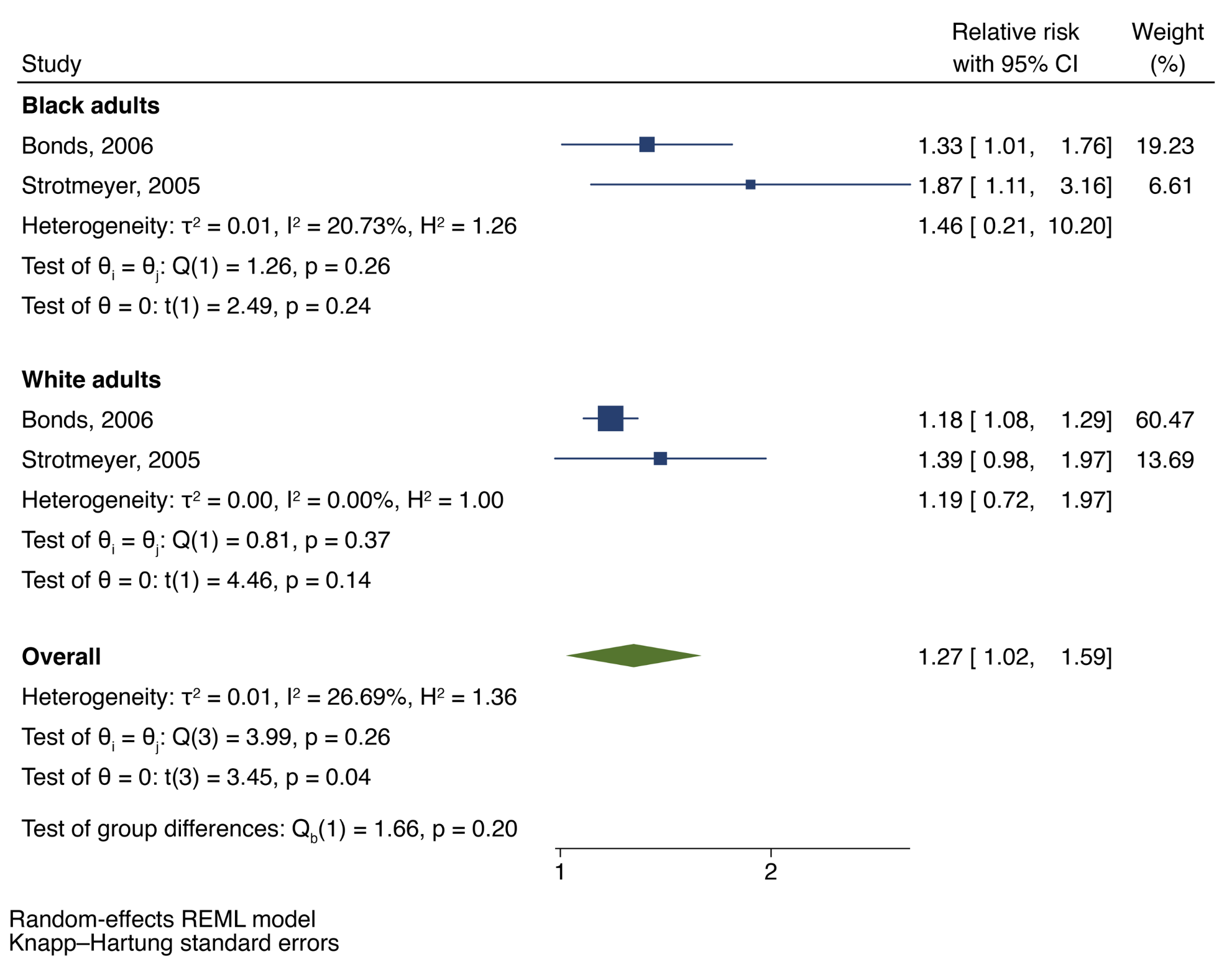
**Supplementary Figure 1. Incident fracture risk (relative risk) in Black adults with DM compared to White adults with DM**
